# A Universal Antigen-Ranking Method to Design Personalized Vaccines Targeting Neoantigens against Melanoma

**DOI:** 10.3390/life13010155

**Published:** 2023-01-05

**Authors:** Iker Malaina, Luis Martínez, Juan Manuel Montoya, Santos Alonso, María Dolores Boyano, Aintzane Asumendi, Rosa Izu, Ana Sanchez-Diez, Goikoane Cancho-Galan, Ildefonso M. de la Fuente

**Affiliations:** 1 Department of Mathematics, University of the Basque Country UPV/EHU, 48940 Bizkaia, Spain; 2 Faculty of Basic Sciences, University of Pamplona, Pamplona 6800, Colombia; 3 Department of Genetics, Physical Anthropology and Animal Physiology, University of the Basque Country UPV/EHU, 48940 Bizkaia, Spain; 4 Department of Cell Biology and Histology, University of the Basque Country UPV/EHU, 48940 Bizkaia, Spain; 5 Biocruces Bizkaia Health Research Institute, 48903 Bizkaia, Spain; 6 Department of Dermatology, Basurto University Hospital, 48013 Bizkaia, Spain; 7 Department of Pathology, Basurto University Hospital, 48013 Bizkaia, Spain; 8 CEBAS-CSIC Institute, Department of Nutrition, 30100 Murcia, Spain

**Keywords:** vaccine design, melanoma, combinatorial optimization, immunogenicity, bioinformatics, personalized medicine

## Abstract

**Background**: The main purpose of this article is to introduce a universal mathematics-aided vaccine design method against malignant melanoma based on neoantigens. The universal method can be adapted to the mutanome of each patient so that a specific candidate vaccine can be tailored for the corresponding patient. **Methods**: We extracted the 1134 most frequent mutations in melanoma, and we associated each of them to a vector with 10 components estimated with different bioinformatics tools, for which we found an aggregated value according to a set of weights, and then we ordered them in decreasing order of the scores. **Results**: We prepared a universal table of the most frequent mutations in melanoma ordered in decreasing order of viability to be used as candidate vaccines, so that the selection of a set of appropriate peptides for each particular patient can be easily and quickly implemented according to their specific mutanome and transcription profile. **Conclusions**: We have shown that the techniques that are commonly used for the design of personalized anti-tumor vaccines against malignant melanoma can be adapted for the design of universal rankings of neoantigens that originate personalized vaccines when the mutanome and transcription profile of specific patients is considered, with the consequent savings in time and money, shortening the design and production time.

## 1. Introduction

Neoantigens arising from mutations in the tumor tissue of patients suffering from different types of cancer are especially appropriate targets to be recognized by the immune system, as they are specific to the affected area. They are usually highly immunogenic as they are not expressed in normal tissues [1]. Neoantigens play an increasingly important role in immunotherapy [2,3,4], and in particular they are used in the design of vaccines aimed to fight several types of cancer, such as kidney cancer [5], glioblastoma [6], non-small cell lung cancer [7], pancreatic cancer [8], hepatocellular carcinoma [9], colon cancer [10], esophageal cancer [11], or breast cancer [12].

In particular, neoantigens have been used to develop personalized vaccines that have given satisfactory results against melanoma [13,14,15,16]. Melanoma has been chosen as the target for a lot of cancer vaccine designs because it is one of the cancers with highest mutation prevalence [17]. Therefore, this kind of tumor presents high immunogenicity, and offers a wide range of possible neoantigens to act against. In addition, the prevalence of this type of cancer is continuously growing, and only in the United States, 7000 deaths have been attributed to it in 2021 [18].

Nowadays, it is neither economically nor humanly possible to experimentally evaluate the individual properties of all possible neoantigens when we are planning to develop a personalized anti-tumor vaccine. To solve this issue, during the last decades, scientists have developed several tools to estimate the main characteristics of peptides [19,20,21,22,23,24]. With the use of these methods, we can perform the in silico phase, prior to synthesizing any peptide, which can identify sequences with potential to be a part of an effective vaccine. As computational neoantigen prediction is an emerging field, besides tools for predicting neoantigens, several methods have been developed to validate those peptides, such as targeted validation and quantification methods [25] or profiling-based identification methods [26].

However, even with these techniques, developing a personalized vaccine for every patient still remains a complex task. In order to ease and generalize this process, in this study, we have analyzed more than a thousand of the most frequent mutations of skin melanoma, and more precisely, the characteristics of the potential neoantigens including such mutations. Those peptides have been ranked according to their potentiality from best to worst so that when anyone intends to develop a personalized vaccine, they only need to select, one by one from the top, those neoantigens for which the corresponding mutation is present in the patient’s tumor and is sufficiently expressed, until a predefined number of neoantigens to be included in the vaccine is obtained. Thus, this work intends to skip the entire process of evaluating and ranking the mutations of each individual, speeding up the process, and giving a universally useful list of potential peptides that can be used to develop efficient anti-melanoma vaccines.

## 2. Materials and Methods

### 2.1. Neoantigen Extraction

We explored the NIH NCI’s (NCancer Institute’s) Genomic Data Commons Data Portal [27] in search for somatic mutations observed in melanoma biopsies that we could use as targets for our vaccine design strategy. We used the following search parameters (as of 7 July 2022):

- Disease type IS nevi and melanomas;
- Primary site IS skin;
- Program name IN(GENIE TCGA);
- Sample type IN (metastatic primary tumor).

In addition, we selected the mutations whose frequencies were at least 0.64%, obtaining 1134 mutations.

Then, we then used the ensembldb package [28] of the Bioconductor [29] open-source software for bioinformatics in order to select the mutations for which all the transcripts in the neighborhood of the mutated position give the same peptide of length 15 after the translation, that is, the mutations giving a unique peptide of length 15 centered in the amino acid corresponding to the mutation. After subselection using ensembldb was concluded, we obtained a set of 896 peptides.

### 2.2. Bioinformatics Tools

For this study, we have used some of the most commonly estimated properties and tools to weight our peptides:

1. T cell class I immunogenicity predictor [30,31]: this tool analyzes the composition and order of the amino acids of the peptide and gives an estimation of the capacity of generating an immunological response of class I T-cells. It has been validated for 9-mers but can be used to study larger peptides. A higher score indicates a higher probability of generating a strong immune response.
2. MHC class I and II binding predictors [32,33,34,35]: these tools determine the ability of the selected peptide to bind a specific MHC molecule. The IEDB website considers several different tools to estimate these properties and returns a set of estimations. However, in order to maintain the same criterion for class I and class II peptides, in this work, we have used the NetMHCpan 4.1 method to estimate the binding affinity for both molecules. As in the immunogenicity prediction tool, higher scores are related to more probable reactions, and in this case, more probable binders. Because both the peptide and the HLA molecule are needed to estimate it, this kind of tool is especially useful for developing personalized vaccines.
3. Gravy Index [36]: The grand average of hydropathicity index (GRAVY) is used to estimate the hydrophobicity of a given amino acid string and is calculated as the average of the hydrophobicity of the individual residues forming the peptide. Positive values suggest that the string will be hydrophobic, whereas negative values indicate that it will be hydrophilic.
4. VaxiJen 2.0 [37]: this tool estimates if a given peptide string is going to react as a potential antigen, and in particular, if it is going to react as a tumor antigen. It sets a threshold of 0.5 as its score, where higher values are expected to react as potential antigens, and values below are more unlikely to act as so.

Notice that because neoantigens are predominantly presented by MHC class I, we have included two variables related to it (T cell class I immunogenicity predictor and MHC class I binding predictor), in contrast to MHC class II, for which we have included only the MHC class II binding predictor.

### 2.3. Weighting the Neoantigens

**Figure 1:**
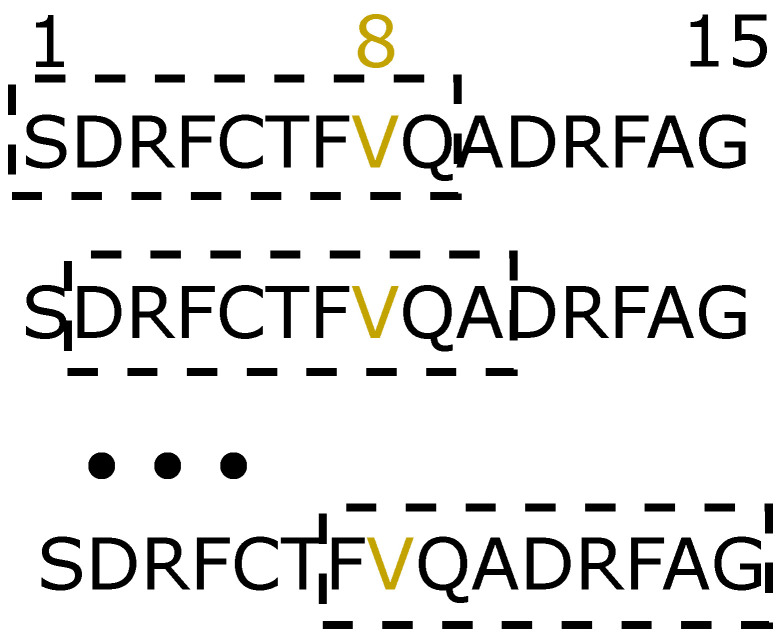
Sliding window to extract shorter strings. In this figure, we illustrate how our sliding window of length 9 applied to 15 amino acid length peptides gives us the seven sub-peptides. In yellow, the position of the mutated amino acid, which is always preserved inside the sub-peptides.

To achieve the objective of ranking the best potential neoantigens, we have applied the tools of the previous subsection to estimate the properties of our strings. However, as we are interested in highlighting the neoantigens that present better characteristics than their non-mutated versions, we used both the values from the neoantigens and the increment between the scores of the neoantigen and the non-mutated string. More precisely, the value for each characteristic was obtained as follows:

1. T cell class I immunogenicity. First, we estimated this characteristic by the IEDB tool described above, for each 15 amino acid string, both mutated and non-mutated. Then, we calculated the subtraction between the neoantigen score and the value of the corresponding non-mutated peptide. Finally, for each string (*i*) we normalized both the neoantigen score ($Im_{i}$) and the subtraction between the neoantigen and the non-mutated string values ($IncIm_{i}$) between 0 and 1.
2. MHC class II binding. As for the previous characteristic, we estimated the value for both neoantigen and non-mutated groups and performed the subtraction between their scores. However, in order to obtain a binding affinity estimation, we need to select the HLA molecule first. In this case, we chose the set of the most representative set of HLA alleles in the population (DRB1*03:01, DRB1*07:01, DRB1*15:01, DRB3*01:01, DRB3*02:02, DRB4*01:01, DRB5*01:01) [38]. Finally, for each neoantigen, as we obtained seven values (one for each molecule), we calculated the average of the corresponding scores and normalized the raw HLA-II score for the mutated string ($HLAII_{i}$) and the increment between the neoantigen and the non-mutated score ($IncHLAII_{i}$) between 0 and 1.
3. MHC class I binding. For this estimation, because the tool recommends its use for strings of length nine, we performed a sliding window to extract the seven strings composing a neoantigen (or non-mutated string). This way, the mutated amino acid (located in the eighth position) is always kept inside the sub-peptides (see Figure 1). Then, for each mutated and non-mutated sub-strings, we calculated the class I binding affinity for the IEDB reference allele set, composed of 27 HLA molecules (A*01:01, A*02:01, A*02:03, A*02:06, A*03:01, A*11:01, A*23:01, A*24:02, A*26:01, A*30:01, A*30:02, A*31:01, A*32:01, A*33:01, A*68:01, A*68:02, B*07:02, B*08:01, B*15:01, B*35:01, B*40:01, B*44:02, B*44:03, B*51:01, B*53:01, B*57:01, B*58:01) [39]. Next, for each neoantigen, we calculated the average of the estimations of the corresponding substrings for every HLA molecule, therefore obtaining a single value for each peptide. Finally, we performed the subtraction between mutated and non-mutated scores as previously conducted for class II molecules. We then normalized those scores between 0 and 1, obtaining two variables, $HLAI_{i}$ and $IncHLAI_{i}$.
4. Gravy index. Because this index is positive for hydrophobic strings, but we are interested in more exposed peptides (i.e., more hydrophilic, which correspond to negative values), after obtaining the score for each string, we normalized the score between 0 and 1 and inverted the order by calculating 1 minus the normalized score (resulting in the variable $Gra_{i}$). Next, as before, we performed the subtraction between the mutated and non-mutated score, obtaining $IncGra_{i}$.
5. VaxiJen score. As has been explained for previous characteristics, we normalized the VaxiJen score between 0 and 1 ($Vax_{i}$) and then performed the subtraction between mutated and non-mutated string values ($IncVax_{i}$).

As a consequence, for each neoantigen, we have obtained a ten-dimensional vector with values between 0 and 1.

### 2.4. Optimization Procedure

In order to combine the aforementioned 10 values for each neoantigen, first, as the immune system is going to act only against the mutated string, we established that the specific values of the neoantigens should be highlighted over the increments, and therefore, we weighted those characteristics double than the increments. This means that the weights of the main characteristics (Im,HLAI,HLAII,Gra and Vax) were multiplied by 2, whereas the increments were multiplied by 1. Next, in order to obtain a normalized amount between 0 and 1, the characteristics were divided by the total (i.e., by 15: the five characteristics weighted double Im,HLAI,HLAII,Gra and Vax, and five increments Inc, therefore 10+5=15). Thus, the final score for each neoantigen ($Score_{i}$) was obtained as follows:

$$Score_{i}=\frac{2}{15}Im_{i}+\frac{2}{15}HLAI_{i}+\frac{2}{15}HLAII_{i}+\frac{2}{15}Gra_{i}+\frac{2}{15}Vax_{i}+\frac{1}{15}+IncIm_{i}+\;+\frac{1}{15}IncHLAI_{i}+\frac{1}{15}IncHLAII_{i}+\frac{1}{15}IncGra_{i}+\frac{1}{15}IncVax_{i}$$

## 3. Results

For obtaining the list of potential neoantigens to develop personalized anti-melanoma vaccines, we first obtained a set of the most frequent 896 mutations in this cancer. Then, the amino acid sequences corresponding to the peptide including those mutations were identified and stored with their corresponding non-mutated version (Section 2.1). Afterwards, we estimated several properties of both the neoantigen and the non-mutated string (Section 2.3), and finally, we combined their scores (Section 2.4) and achieved an ordination of the neoantigens according to their characteristics. The first 30 elements of the list are displayed in Table 1; for the full ranking, see Supplementary Materials, Table S1.

**Figure 2:**
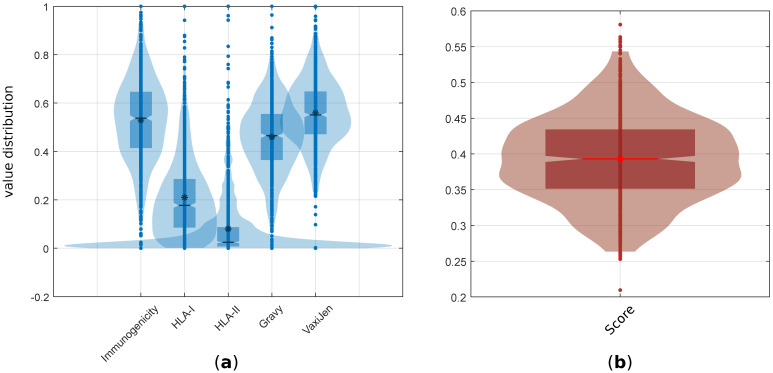
Violin plots for the value distributions. (**a**) The distribution of the five main characteristics (immunogenicity, HLA-I binding, HLA-II binding, Gravy index, and VaxiJen score). (**b**) In this panel, we depict the distribution of the combined score variable. The dots indicate individual values, * is the average value, the central box covers the values from Q3 to Q1, and the horizontal bar indicates the median value.

The distribution of the values varied depending on the variable. We observed that immunogenicity, the Gravy index, and the VaxiJen score were normally distributed (*p*-values: 0.722, 0.605 and 0.184, respectively, as determined via the Kolmogorov–Smirnov test), whereas the HLA binding variables were not (*p*-values: 10${}^{-9}$ and $10^{-61}$ for HLA-I and HLA-II, respectively). In Figure 2, we represent the distribution and main descriptive variables in a violin plot [40] for (**a**) the main five characteristics of the neoantigens, and (**b**), the final score.

The highest score value was 0.5809, obtained by the neoantigen YRKLTVEENYRIEEE (mutation: chr7:g.143756443C>T), whereas the lowest was 0.2096, achieved by VEGQQLVRPKKLPLI (mutation: chr8:g.24442511C>T).

The highest values of individual characteristics of the neoantigens (because they were normalized, this value was set to 1) were the following: YNFISIFSFLEIWYT (chr1:g.158717916G>A) achieved the highest immunogenicity score, DLPSIYPSFTYYRSG (mutation: chr19:g.43175439G>A) achieved the highest HLA-I binding affinity, YRKLTVEENYRIEEE (mutation: chr7:g.143756443C>T) achieved the highest HLA-II binding affinity, KHEDNKQEENKENRK (mutation: chr1:g.152314540C>T) achieved the highest Gravy index, and finally, PRFKRLGELYSVGES (mutation: chr16:g.61653646C>T) achieved the highest VaxiJen score.

Regarding the increments, the highest difference between the mutated and non-mutated immunogenicity score was achieved by the neoantigen EALMSELKVLSYLGN (mutation: chr4:g.54728055A>G). For HLA-I, it was obtained by DAHSVLKRFPRANEF (chrX:g.151700035G>A). For HLA-II, it was obtained by PAIHWISPEGKLISN (mutation: chr14:g.41887592C>T). For the Gravy index, it was obtained by achieved GDFGLATVKSRWSGS (mutation: chr7:g.140753336A>T). Finally, the highest increment for the VaxiJen score was achieved by the neoantigen SPSRPLNGLLRLGLP (mutation: chr3:g.147396359C>T).

## 4. Discussion

In this work, we have performed a study of the main immunological properties for almost a thousand potential neoantigens with the use of bioinformatics. After analyzing the characteristics of mutated and non-mutated peptides, we combined their estimated values and obtained a ranking ordered from the most promising neoantigen to the least promising. The objective of the study is to offer a list of potential neoantigens for designing personalized vaccines, and its use is very simple: after studying the particular mutations of the patient’s tumor, the vaccine developers should go from top to bottom of Table S1, selecting those neoantigens for which the mutations are present in that specific tumor and then use them to design the vaccine.

Due to the increasing interest in neoepitope prediction, several bioinformatic approaches are currently being proposed. For a detailed overview on recent methodologies, see the reviews: [41,42,43,44]. However, all of those works agree on one thing, which is that even if the effectiveness of the techniques looks promising and can improve personalized patient care, predicting neoantigens with all the required computational steps is still a complex issue of discussion.

To date, the methodologies for developing personalized vaccines against cancer have been focused on the specific patients of the study [13,14,15], and therefore, even if the results have been promising, they cannot be applied to new patients, limiting their usefulness.

On the contrary, our work offers a very large list of the most frequent mutations in melanoma and a quantitative analysis of their potentiality. Therefore, we consider that this study can be of great help for future immunologists, but more importantly, can give new hope to cancer patients by speeding up and simplifying the process of personalized anti-tumor vaccine design.

Notice that there are several interesting characteristics of neoepitopes, such as IFN gamma production, humoral immunity stimulation, allergenicity, or docking evaluation, which could also be considered in order to improve our methodology, and in general, the bioinformatic prediction of peptides. However, in this work, we have chosen the most widely used characteristics, firstly, because their use is more extended and tested, and secondly, because the inclusion of more variables shrinks the effect of the other characteristics in the objective function.

It has to be mentioned that even if the study has been performed by choosing the 896 most frequent mutations of skin melanoma, the list can be also used for other types of cancer (due to the sharing of mutations [45]) following the same selection procedure. Although it would be less cancer specific, it might also give good results because the estimated bioinformatics characteristics did not consider the type of cancer, and therefore, the ordering is not cancer dependent (despite the initial selection of the most frequent mutations).

Finally, as future work, our objective is to use this list to experimentally develop and test anti-melanoma personalized vaccines for several patients, validating its value.

## Figures and Tables

**Table 1:** Best 30 scoring potential neoantigens.

Mutation	Non-Mutated	Neoantigen	Score
chr7:g.143756443C>T	YRKLTVEENYRIEEE	YRKLTVEKNYRIEEE	0.5809
chr15:g.71728609G>A	NWAIDRPGKYEGGGT	NWAIDRPEKYEGGGT	0.5635
chr7:g.87544253G>A	DESIPPVSFWRIMKL	DESIPPVFFWRIMKL	0.5605
chr5:g.26881525C>T	DNIVTYNDEGGGEED	DNIVTYNNEGGGEED	0.5577
chr19:g.43175439G>A	DLPSIYPSFTYYRSG	DLPSIYPLFTYYRSG	0.5560
chrX:g.1309468G>A	RRGLDREGNYLRPRG	RRGLDREENYLRPRG	0.5522
chr7:g.82952797C>T	KPQYKEDGKLQLVGD	KPQYKEDEKLQLVGD	0.5510
chr5:g.26885776C>T	PILIFDNDYPIQSST	PILIFDNNYPIQSST	0.5495
chr14:g.19827570G>A	FYFIILPGNFLIIFT	FYFIILPENFLIIFT	0.5450
chr19:g.43184859C>T	NVTREDAGSYTLHII	NVTREDAESYTLHII	0.5416
chrX:g.151700035G>A	DAHSVLKRFPRANEF	DAHSVLKQFPRANEF	0.5402
chrX:g.50194060G>A	ECSIDDLSFYVNRLS	ECSIDDLFFYVNRLS	0.5329
chr2:g.154259080G>A	RNYFEEIGTYDAGMD	RNYFEEIETYDAGMD	0.5298
chr12:g.101677299G>A	TITELVIGNEYYFRV	TITELVIENEYYFRV	0.5292
chr17:g.40770092C>T	VLQYTAGGNVNVEMN	VLQYTAGRNVNVEMN	0.5258
chrX:g.3317733G>A	GTPAPQISWIFPDRR	GTPAPQIFWIFPDRR	0.5255
chr18:g.31391183G>A	IKVLDVNDNFPTLEK	IKVLDVNNNFPTLEK	0.5230
chr3:g.36832438C>T	GRGSRIKGIEGKFGM	GRGSRIKEIEGKFGM	0.5219
chr18:g.31334141G>A	FFISGNEGNWFEIEM	FFISGNEENWFEIEM	0.5197
chr22:g.38488052T>C	NSSEDYVHRIGRTAR	NSSEDYVRRIGRTAR	0.5188
chr2:g.197402759C>T	DNMDEYVRNTTARAF	DNMDEYVHNTTARAF	0.5177
chrX:g.123404754G>A	KNHEQLEGNERYEGY	KNHEQLEENERYEGY	0.5161
chr7:g.148415509G>A	KGTYHTNEAKGAESA	KGTYHTNKAKGAESA	0.5155
chr1:g.175393814G>A	QWEPFSFSFDGWEIS	QWEPFSFFFDGWEIS	0.5118
chr16:g.69931192C>T	GAYDRSFRWKYHQFR	GAYDRSFWWKYHQFR	0.5113
chr7:g.142751821G>A	HNIEVLEGNEQFINA	HNIEVLEENEQFINA	0.5109
chr1:g.233160330G>A	HHPWMWISHPILKNK	HHPWMWILHPILKNK	0.5109
chr2:g.191836347C>T	NHQKISSGKSSPFKV	NHQKISSEKSSPFKV	0.5102
chr6:g.69324972G>A	QSYMAVTGKIRTRLI	QSYMAVTEKIRTRLI	0.5102
chr18:g.31522149C>T	NTLNSKISYRIVSLE	NTLNSKIFYRIVSLE	0.5078

## Data Availability

Not applicable.

## Supplementary Materials

The following supporting information can be downloaded at: https://www.mdpi.com/article/10.3390/life13010155/s1, Table S1: the full table for the best scoring 896 potential neoantigen ranking.

## Abbreviations

The following abbreviations are used in this manuscript:

MHC	Major Histocompatibility Complex
