# Supplementary material for: A Universal Antigen-Ranking Method to Design Personalized Vaccines Targeting Neoantigens against Melanoma
Source: Life (Basel). 2023 Jan 5;13(1):155. doi: 10.3390/life13010155 (PMC9867041; doi:10.3390/life13010155)
Supplement: Supplementary file 1 [file life-13-00155-s001.zip › life-2031973-supplementary.pdf]

Supplementary Material

Table S1. Full table for the best scoring potential neoatigen ranking.

Mutation	Non-mutated	Neoantigen	Score
**chr7:g.143756443C>T**	YRKLTVEENYRIEEE	YRKLTVEKNYRIEEE	0,5809
**chr15:g.71728609G>A**	NWAIDRPGKYEGGGT	NWAIDRPEKYEGGGT	0,5635
**chr7:g.87544253G>A**	DESIPPVSFWRIMKL	DESIPPVFFWRIMKL	0,5605
**chr5:g.26881525C>T**	DNIVTYNDEGGGEED	DNIVTYNNEGGGEED	0,5577
**chr19:g.43175439G>A**	DLPSIYPSFTYYRSG	DLPSIYPLFTYYRSG	0,556
**chrX:g.1309468G>A**	RRGLDREGNYLRPRG	RRGLDREENYLRPRG	0,5522
**chr7:g.82952797C>T**	KPQYKEDGKLQLVGD	KPQYKEDEKLQLVGD	0,551
**chr5:g.26885776C>T**	PILIFDNDYPIQSST	PILIFDNNYPIQSST	0,5495
**chr14:g.19827570G>A**	FYFIILPGNFLIIFT	FYFIILPENFLIIFT	0,545
**chr19:g.43184859C>T**	NVTREDAGSYTLHII	NVTREDAESYTLHII	0,5416
**chrX:g.151700035G>A**	DAHSVLKRFPRANEF	DAHSVLKQFPRANEF	0,5402
**chrX:g.50194060G>A**	ECSIDDLSFYVNRLS	ECSIDDLFFYVNRLS	0,5329
**chr2:g.154259080G>A**	RNYFEEIGTYDAGMD	RNYFEEIETYDAGMD	0,5298
**chr12:g.101677299G>A**	TITELVIGNEYYFRV	TITELVIENEYYFRV	0,5292
**chr17:g.40770092C>T**	VLQYTAGGNVNVEMN	VLQYTAGRNVNVEMN	0,5258
**chrX:g.3317733G>A**	GTPAPQISWIFPDRR	GTPAPQIFWIFPDRR	0,5255
**chr18:g.31391183G>A**	IKVLDVNDNFPTLEK	IKVLDVNNNFPTLEK	0,523
**chr3:g.36832438C>T**	GRGSRIKGIEGKFGM	GRGSRIKEIEGKFGM	0,5219
**chr18:g.31334141G>A**	FFISGNEGNWFEIEM	FFISGNEENWFEIEM	0,5197
**chr22:g.38488052T>C**	NSSEDYVHRIGRTAR	NSSEDYVRRIGRTAR	0,5188
**chr2:g.197402759C>T**	DNMDEYVRNTTARAF	DNMDEYVHNTTARAF	0,5177
**chrX:g.123404754G>A**	KNHEQLEGNERYEGY	KNHEQLEENERYEGY	0,5161
**chr7:g.148415509G>A**	KGTYHTNEAKGAESA	KGTYHTNKAKGAESA	0,5155
**chr1:g.175393814G>A**	QWEPFSFSFDGWEIS	QWEPFSFFFDGWEIS	0,5118
**chr16:g.69931192C>T**	GAYDRSFRWKYHQFR	GAYDRSFWWKYHQFR	0,5113
**chr7:g.142751821G>A**	HNIEVLEGNEQFINA	HNIEVLEENEQFINA	0,5109
**chr1:g.233160330G>A**	HHPWMWISHPILKNK	HHPWMWILHPILKNK	0,5109
**chr2:g.191836347C>T**	NHQKISSGKSSPFKV	NHQKISSEKSSPFKV	0,5102
**chr6:g.69324972G>A**	QSYMAVTGKIRTRLI	QSYMAVTEKIRTRLI	0,5102
**chr18:g.31522149C>T**	NTLNSKISYRIVSLE	NTLNSKIFYRIVSLE	0,5078
**chr8:g.24485306G>A**	WLYSHVQGISYPGGM	WLYSHVQEISYPGGM	0,5035
**chrX:g.71291940C>T**	KAGEVFIHKDKGFGF	KAGEVFIYKDKGFGF	0,5034
**chr3:g.98149867G>A**	LHALIHEGFLFRLTF	LHALIHEEFLFRLTF	0,5021
**chr10:g.24524613C>T**	NLPHVASSPAVPQEA	NLPHVASFPAVPQEA	0,5004
**chrX:g.106036667C>T**	KELELQIGNALFIGK	KELELQIENALFIGK	0,5004
**chr1:g.47067629C>T**	IRALHLFPAPPAHWF	IRALHLFSAPPAHWF	0,5001
**chr6:g.152539997C>T**	EQEIVQKRTFTKWIN	EQEIVQKQTFTKWIN	0,4988
**chr8:g.86229637C>T**	LIQAIFQGYFAYSGG	LIQAIFQEYFAYSGG	0,498
**chr18:g.72858855C>T**	DGELESMGFSARYNF	DGELESMEFSARYNF	0,4975

**chr1:g.182665995G>A**	EATRWADSFDVLLSH	EATRWADFFDVLLSH	0,4972
**chr1:g.118016053C>T**	WFTTTPEGNRIGTKG	WFTTTPEENRIGTKG	0,4968
**chr2:g.27232614C>T**	RTPHVLVLGSGVYRI	RTPHVLVFGSGVYRI	0,4966
**chr5:g.96410946C>T**	SIFVWASGNGGRQGD	SIFVWASENGGRQGD	0,4965
**chr2:g.50531346G>A**	HTEAEDVSLRFRSQR	HTEAEDVFLRFRSQR	0,4957
**chr2:g.140716021G>A**	SFRYVIISQGLDQPR	SFRYVIIFQGLDQPR	0,4951
**chr2:g.184933637G>A**	LKQREFARNVASKSR	LKQREFAQNVASKSR	0,4951
**chr3:g.147396359C>T**	SPSRPLNGLLRLGLP	SPSRPLNRLLRLGLP	0,4941
**chr8:g.14554847C>T**	NAQLYPVGIYGWRKR	NAQLYPVEIYGWRKR	0,4926
**chr8:g.123980648C>T**	RAQESKFSKALKELK	RAQESKFFKALKELK	0,4924
**chrX:g.3317665C>T**	EGRITLHENRTLSIK	EGRITLHKNRTLSIK	0,4921
**chr13:g.28712799C>T**	SFLTSFLGIWLFSYC	SFLTSFLEIWLFSYC	0,4917
**chr5:g.24487811C>T**	LATYAYEGNDSIAES	LATYAYEENDSIAES	0,4916
**chr11:g.92798561C>T**	TIAHFHFHVHVRDSG	TIAHFHFYVHVRDSG	0,4914
**chr5:g.41018960G>A**	NILEENIRRLLPLPP	NILEENIWRLLPLPP	0,4913
**chrX:g.3330248C>T**	LRLLHLEGNLLHQLH	LRLLHLEENLLHQLH	0,4904
**chr16:g.77320027G>A**	EIQELQVSSSYLAVR	EIQELQVFSSYLAVR	0,4899
**chr16:g.21201993C>T**	DKVVYENEIHALWTD	DKVVYENKIHALWTD	0,4896
**chr6:g.55255187C>T**	LSCIALDRWYAICHP	LSCIALDWWYAICHP	0,4885
**chr15:g.57438828C>T**	KTRPDVLPFRRQDSA	KTRPDVLSFRRQDSA	0,4882
**chr1:g.233666883C>T**	DDWNFLESFYFCFIS	DDWNFLEFFYFCFIS	0,488
**chr1:g.97699379C>T**	YSDITIFEKQEYVGG	YSDITIFKKQEYVGG	0,4879
**chr12:g.96742482C>T**	SEFLDPISLNAREYF	SEFLDPIFLNAREYF	0,4869
**chr8:g.130780658C>T**	KGISEQEGKIKTYFL	KGISEQEEKIKTYFL	0,4867
**chr4:g.154584191G>A**	ENGVVWVSFRGADYS	ENGVVWVFFRGADYS	0,4865
**chr20:g.1571020C>T**	QLTWLENGNVSRTET	QLTWLENENVSRTET	0,4846
**chr9:g.14863876C>T**	DCHFLPNEVKYVHNG	DCHFLPNKVKYVHNG	0,4843
**chr8:g.109408124G>A**	DITEQTKGKPNLETF	DITEQTKEKPNLETF	0,4842
**chrX:g.152255974G>A**	YTIDVFFRQTWHDER	YTIDVFFWQTWHDER	0,4832
**chr8:g.3367061G>A**	FISDFSISYEGFNIT	FISDFSILYEGFNIT	0,4816
**chr2:g.230037495G>A**	ITQKYDFSFYICGLL	ITQKYDFFFYICGLL	0,4807
**chr4:g.74449137G>A**	KKKGGKNGKNRRNRK	KKKGGKNEKNRRNRK	0,4805
**chr1:g.247605707G>A**	FYLLTLVGNFTIIII	FYLLTLVENFTIIII	0,4798
**chr14:g.33560158G>A**	VFALNQEGKFLYISE	VFALNQEEKFLYISE	0,4795
**chr3:g.169093015C>T**	DAYFTEIRNFIGNSN	DAYFTEIQNFIGNSN	0,4784
**chr13:g.91693300G>A**	SVQEFFTDVGLYLFG	SVQEFFTNVGLYLFG	0,477
**chr4:g.54728055A>G**	EALMSELKVLSYLGN	EALMSELEVLSYLGN	0,4768
**chr7:g.142752910G>A**	VCNGQLQGVVSWGDG	VCNGQLQRVVSWGDG	0,4765
**chr11:g.58834539G>A**	KEIPFYFHVADNNEK	KEIPFYFYVADNNEK	0,4762
**chr20:g.42161391C>T**	LLQHITQMKRGQGYG	LLQHITQIKRGQGYG	0,4756
**chr17:g.10497154C>T**	TEQIAEGGKRIHELE	TEQIAEGEKRIHELE	0,4753
**chr4:g.1728651C>T**	MDDPNFIPFGGDTKS	MDDPNFISFGGDTKS	0,4752
**chr2:g.135808524C>T**	PIYITENGVGLTNPN	PIYITENRVGLTNPN	0,475

**chr1:g.158355902G>A**	QLVCHVSGFYPKPVW	QLVCHVSEFYPKPVW	0,4749
**chr7:g.87545921C>T**	DGVIVEKGNHDELMK	DGVIVEKENHDELMK	0,4748
**chr17:g.10492520C>T**	KFRRIQHELEEAEER	KFRRIQHKLEEAEER	0,4747
**chr5:g.179158774C>T**	TGHDEYHDHAIFLTR	TGHDEYHNHAIFLTR	0,4743
**chr5:g.39134887C>T**	RITDNPEGKWLGRTA	RITDNPEEKWLGRTA	0,4734
**chrX:g.141897520G>A**	REHFIYGDPRKLLTI	REHFIYGNPRKLLTI	0,4718
**chr19:g.56516312C>T**	KDVAIDFSQEEWQWM	KDVAIDFFQEEWQWM	0,4717
**chr17:g.4937836G>A**	SLWKGFTPYYARLGP	SLWKGFTSYYARLGP	0,4711
**chr20:g.1636379C>T**	TLKWFKNGNELSDFQ	TLKWFKNENELSDFQ	0,4708
**chr11:g.76351127G>A**	LPDIKFFPNVYALLK	LPDIKFFSNVYALLK	0,4704
**chr8:g.105802180G>A**	NITFSRHETYMVHKQ	NITFSRHKTYMVHKQ	0,4695
**chr19:g.22757311G>A**	LRKHEIIHTGKKPYK	LRKHEIIYTGKKPYK	0,4695
**chr5:g.94953325C>T**	YVKFKIGGKEVFRSK	YVKFKIGEKEVFRSK	0,4685
**chr10:g.60263938C>T**	LLENDTKGKVRLPAL	LLENDTKEKVRLPAL	0,4682
**chr1:g.217683288C>T**	RWRGYRVRKYLFNYY	RWRGYRVWKYLFNYY	0,4669
**chr17:g.11822463G>A**	TLCFSPVGNKLRVRS	TLCFSPVENKLRVRS	0,466
**chr19:g.44428742C>T**	KHQRVHTGKKPYTCD	KHQRVHTEKKPYTCD	0,4658
**chr4:g.128842980C>T**	STTWSQNSRSQHRRS	STTWSQNFRSQHRRS	0,4655
**chr4:g.99342826G>A**	TDGGVDFSFEVIGRL	TDGGVDFLFEVIGRL	0,465
**chr14:g.18601616G>A**	VILCWVCGFLWFLIP	VILCWVCEFLWFLIP	0,4649
**chr11:g.4804110G>A**	MLAIFWFHAHEIQYH	MLAIFWFYAHEIQYH	0,4648
**chr14:g.88186062C>T**	RLSVEIHDKLQRAAT	RLSVEIHNKLQRAAT	0,4648
**chr2:g.218584683C>T**	HTVSKTRPFEYLRLT	HTVSKTRLFEYLRLT	0,4647
**chr14:g.19920797G>A**	SPMYFLLGNLSFVDI	SPMYFLLENLSFVDI	0,4646
**chr7:g.140781603C>T**	RIGSGSFGTVYKGKW	RIGSGSFRTVYKGKW	0,4642
**chr22:g.15528394G>A**	TPMYMFLGNFSFLEI	TPMYMFLENFSFLEI	0,4639
**chr8:g.54622262G>A**	SQRVYPKGNAKSESR	SQRVYPKENAKSESR	0,4624
**chr14:g.41887592C>T**	PAIHWISPEGKLISN	PAIHWISSEGKLISN	0,4623
**chr7:g.142055649G>A**	KYLYGFGETEHRSYR	KYLYGFGKTEHRSYR	0,462
**chr19:g.52438334C>T**	LAQHRKIHTGEKPYK	LAQHRKIYTGEKPYK	0,462
**chr7:g.140753336A>T**	GDFGLATVKSRWSGS	GDFGLATEKSRWSGS	0,4618
**chr3:g.7579262G>A**	GVPENFNEAKPIGFT	GVPENFNKAKPIGFT	0,4617
**chr6:g.130440906G>A**	TKIIHMRDIYSTVID	TKIIHMRNIYSTVID	0,4614
**chr2:g.178782877C>T**	REAFAEDSGRFTCSA	REAFAEDNGRFTCSA	0,4599
**chr8:g.72567790C>T**	KTSRSTLSLPPEPVD	KTSRSTLFLPPEPVD	0,4597
**chr1:g.159307820G>A**	ILFAVDTGLFISTQQ	ILFAVDTELFISTQQ	0,4596
**chr1:g.152760577C>T**	PKCRIEISSPCCPRQ	PKCRIEIFSPCCPRQ	0,4594
**chr16:g.26135994G>A**	GEMAKVQDFLGLKRV	GEMAKVQNFLGLKRV	0,4592
**chr9:g.101370989C>T**	YVAPGVTRIKVREGR	YVAPGVTQIKVREGR	0,4582
**chr6:g.56179883C>T**	ESILYLGGNTKTGKA	ESILYLGENTKTGKA	0,4577
**chr2:g.233682500C>T**	AQCPAPLSYVPRLLL	AQCPAPLFYVPRLLL	0,4574
**chr15:g.40382906C>T**	WLSTECDSHPLPPSY	WLSTECDFHPLPPSY	0,4572
**chr18:g.31089522G>A**	PGVDQEPRNLFYVER	PGVDQEPWNLFYVER	0,4572

**chr11:g.55968222C>T**	MDIWTQKGNISLFAC	MDIWTQKENISLFAC	0,457
**chr2:g.166038050C>T**	GNSVGALGNLTLVLA	GNSVGALENLTLVLA	0,4569
**chr1:g.247574356G>A**	CSCTHEHDYEVVFPH	CSCTHEHNYEVVFPH	0,4564
**chr8:g.109459717G>A**	ITVTLPDGTLFEARA	ITVTLPDETLFEARA	0,4556
**chr20:g.45207407C>T**	GRLPSEFSQFPHGQK	GRLPSEFFQFPHGQK	0,4555
**chr10:g.105147641G>A**	MHIISTDENQVFAAV	MHIISTDKNQVFAAV	0,4555
**chr19:g.22180978G>A**	LTKHKIIHTGEKPYK	LTKHKIIYTGEKPYK	0,4554
**chr1:g.43603456C>T**	LTPETTYSVTVAAYT	LTPETTYFVTVAAYT	0,4553
**chr7:g.82915294G>A**	TSSIGGISSRARLLQ	TSSIGGIFSRARLLQ	0,4549
**chr3:g.193321794G>A**	TLCQFPFSSSLQRMS	TLCQFPFFSSLQRMS	0,4549
**chr5:g.38418229G>A**	DMRPWPLGKALSGAD	DMRPWPLEKALSGAD	0,4547
**chrX:g.66256246G>A**	NKSWYLEENVATHGS	NKSWYLEKNVATHGS	0,4542
**chr14:g.91669852G>A**	SPVGMVFHPRSHFLY	SPVGMVFYPRSHFLY	0,4542
**chr6:g.73077787G>A**	LITAWYIGFLVLIFS	LITAWYIEFLVLIFS	0,454
**chr11:g.100340557G>A**	VILTGLEGNTLYHFT	VILTGLEENTLYHFT	0,4536
**chr4:g.188147302C>T**	EALRPIFSPCLPNEG	EALRPIFFPCLPNEG	0,4535
**chr8:g.39645363G>A**	PDAIGLEGFSVIIAQ	PDAIGLEEFSVIIAQ	0,453
**chr8:g.113019096C>T**	FVTDSNHRYRGFSAP	FVTDSNHQYRGFSAP	0,4527
**chr8:g.51408643C>T**	ALNPHWEGNTVYQEA	ALNPHWEENTVYQEA	0,4519
**chr4:g.176221220C>T**	DQEIPHLGTPLYVAC	DQEIPHLETPLYVAC	0,4519
**chr1:g.74636410C>T**	PRPYTAPGNMQPPIR	PRPYTAPENMQPPIR	0,4516
**chr1:g.13342691G>A**	APEESLNSLVRVDWE	APEESLNFLVRVDWE	0,4512
**chr3:g.98168746G>A**	LTEFVLTGFLYQPQW	LTEFVLTEFLYQPQW	0,4512
**chr4:g.54727444T>C**	MYEVQWKVVEEINGN	MYEVQWKAVEEINGN	0,4511
**chr6:g.7583897G>A**	KRSMSFQGIRQPVTV	KRSMSFQEIRQPVTV	0,4507
**chr14:g.94288397C>T**	VLKLPYQGNATMLVV	VLKLPYQENATMLVV	0,45
**chr9:g.74763034C>T**	SQVGHLQDLSALTVD	SQVGHLQNLSALTVD	0,4496
**chr3:g.131542612C>T**	GILRSPKGEPVLRDI	GILRSPKEEPVLRDI	0,4495
**chr17:g.10526642C>T**	AQWRTKYETDAIQRT	AQWRTKYKTDAIQRT	0,4494
**chr17:g.75729303C>T**	PNSDPPFSFKNVISL	PNSDPPFFFKNVISL	0,4494
**chr2:g.227059518C>T**	PGVNGQKGIPGDPAF	PGVNGQKEIPGDPAF	0,4493
**chr11:g.65605976G>A**	CWAWGPSSPKPGEAQ	CWAWGPSFPKPGEAQ	0,449
**chr1:g.172042115C>T**	AKINRIFHERFPFEI	AKINRIFYERFPFEI	0,4487
**chr2:g.102665135G>A**	LFKSFCQMKTIETID	LFKSFCQIKTIETID	0,4483
**chr8:g.85129207C>T**	IIFRERNSKGQLEVM	IIFRERNFKGQLEVM	0,4476
**chr10:g.49107660C>T**	HYVCRVQEISRHRNK	HYVCRVQKISRHRNK	0,4474
**chr1:g.216519284C>T**	ADDYIMDEDQSKLAG	ADDYIMDKDQSKLAG	0,4469
**chr2:g.227051095C>T**	PGRYGPPGFHRGEPG	PGRYGPPEFHRGEPG	0,4466
**chr13:g.57724776G>A**	NVETETYETVNPTGK	NVETETYKTVNPTGK	0,446
**chr4:g.99345053C>T**	NPRGTLQDGTRRFTC	NPRGTLQNGTRRFTC	0,4457
**chr1:g.158717916G>A**	YNFISIFSFLEIWYT	YNFISIFFFLEIWYT	0,4455
**chr1:g.239908220G>A**	LYWRIYKETEKRTKE	LYWRIYKKTEKRTKE	0,4453
**chr19:g.55858466G>A**	LKQEMQKGKDLALTC	LKQEMQKEKDLALTC	0,4452

**chr10:g.71570862G>A**	ITDVQDMDPIFINLP	ITDVQDMNPIFINLP	0,445
**chr11:g.124117765C>T**	YENEEKVPLEAFFVF	YENEEKVSLEAFFVF	0,4449
**chr3:g.38614022C>T**	LGNLSALRTFRVLRA	LGNLSALQTFRVLRA	0,4449
**chr4:g.68818106C>T**	NSWNFKFPHPFLPNV	NSWNFKFSHPFLPNV	0,4448
**chr16:g.28903404G>A**	LPVIGLDEILKFVAR	LPVIGLDKILKFVAR	0,4448
**chr3:g.161497145G>A**	SPVPGQKGEPGETGQ	SPVPGQKEEPGETGQ	0,4447
**chr7:g.150572210G>A**	GAGKSATGNSILGRK	GAGKSATENSILGRK	0,4442
**chr12:g.18701736C>T**	LVKNKKIGTLKETHE	LVKNKKIETLKETHE	0,4438
**chr10:g.28089731C>T**	TADVPTYEEVTPYRR	TADVPTYKEVTPYRR	0,4437
**chr2:g.178774374C>T**	VSPENIEGKWYHNDV	VSPENIEEKWYHNDV	0,4436
**chr1:g.107760822G>A**	LRDLLVVPMQRVLKY	LRDLLVVSMQRVLKY	0,4429
**chr9:g.116188053G>A**	LTGHDGGDCRHLRHP	LTGHDGGNCRHLRHP	0,4429
**chr11:g.4488989C>T**	EHLHAWISIPFCFAY	EHLHAWIFIPFCFAY	0,4428
**chr1:g.151366370G>A**	SLKDGLIPLEIRFLH	SLKDGLISLEIRFLH	0,4428
**chr3:g.26709910C>T**	SNQITSIPNEIFKDL	SNQITSISNEIFKDL	0,4425
**chr3:g.35792478G>A**	NVINNQQGTPVQSVM	NVINNQQETPVQSVM	0,4421
**chr2:g.187496934C>T**	FIYGGCEGNQNRFES	FIYGGCEENQNRFES	0,4421
**chr8:g.92011064G>A**	TLTIEEFHSKLQEAT	TLTIEEFYSKLQEAT	0,4419
**chr7:g.140753336A>C**	GDFGLATVKSRWSGS	GDFGLATGKSRWSGS	0,4415
**chr1:g.117442270C>T**	VQLAEKLLPAFNTPT	VQLAEKLFPAFNTPT	0,4415
**chr4:g.166003417G>A**	CSYVGRRGNGPQAIS	CSYVGRRENGPQAIS	0,4415
**chr1:g.118885415G>A**	SCSPPTFHLAPNTFN	SCSPPTFYLAPNTFN	0,4415
**chr1:g.56881485G>A**	EDAQSVYDASYYGGQ	EDAQSVYNASYYGGQ	0,4415
**chr3:g.57109588G>A**	FVKVVPFPSIKNESN	FVKVVPFSSIKNESN	0,4412
**chr11:g.19056263C>T**	IALVGLVGNGFVLWL	IALVGLVENGFVLWL	0,4412
**chr1:g.157524435G>A**	PILYWFYHEDVTLGK	PILYWFYYEDVTLGK	0,4411
**chr5:g.31299560G>A**	DMGGQMGGLSGTTTV	DMGGQMGELSGTTTV	0,4409
**chr10:g.45789214C>T**	STGTGSQSVERTKPK	STGTGSQFVERTKPK	0,4406
**chr3:g.98007862C>T**	LMLYWKHGNKSLNTE	LMLYWKHENKSLNTE	0,4406
**chr20:g.42315779G>A**	NGYWNPPLSPLKSYS	NGYWNPPFSPLKSYS	0,4405
**chr16:g.51141996C>T**	QLVLIVNENPASPPE	QLVLIVNKNPASPPE	0,4405
**chr1:g.176594599G>A**	IRSGKDKGKRDARFF	IRSGKDKEKRDARFF	0,4404
**chr17:g.62737146C>T**	ALSQAFQGKNSPQVL	ALSQAFQEKNSPQVL	0,4402
**chr6:g.167340735C>T**	VYQEGLVRFATEKFD	VYQEGLVWFATEKFD	0,4401
**chr7:g.140781611C>T**	VGQRIGSGSFGTVYK	VGQRIGSESFGTVYK	0,4397
**chr3:g.169381284C>T**	NMPGAGLGIWTKRKI	NMPGAGLEIWTKRKI	0,4396
**chr1:g.159440635C>T**	VTYTVITPLLNPVVY	VTYTVITSLLNPVVY	0,4395
**chr14:g.19781178C>T**	VIFPLLNPIIYTLRN	VIFPLLNSIIYTLRN	0,4389
**chr5:g.146000175G>A**	DENWYQGEINGISGN	DENWYQGKINGISGN	0,4387
**chr1:g.248626488G>A**	PISIISTSYSLILLT	PISIISTFYSLILLT	0,4386
**chr4:g.23828505C>T**	LKYLTTNDDPPHTKP	LKYLTTNNDPPHTKP	0,4386
**chr8:g.2949322C>T**	DIHGKDFGKFKLERQ	DIHGKDFEKFKLERQ	0,4376
**chr3:g.123733717C>T**	PQSQEVKENQTVKFR	PQSQEVKKNQTVKFR	0,4375

**chr4:g.157152784C>T**	AAELPKVSYVKALDV	AAELPKVFYVKALDV	0,437
**chr3:g.114295787G>A**	LQSLTVNDTGEYFCI	LQSLTVNNTGEYFCI	0,4367
**chr2:g.227273055G>A**	PPGYGPQGEPGLQGT	PPGYGPQEEPGLQGT	0,4365
**chr15:g.91226393G>A**	EGHDEEDEIYEGEYQ	EGHDEEDKIYEGEYQ	0,4365
**chr15:g.71898605G>A**	RSLGGISPSEDRRWS	RSLGGISSSEDRRWS	0,4364
**chr19:g.21972399G>A**	WPSTLSYHKKIHTGE	WPSTLSYYKKIHTGE	0,4358
**chr11:g.100255892C>T**	LQARSPFSLGWQTVK	LQARSPFFLGWQTVK	0,4354
**chr8:g.3348137C>T**	ECGASVKGNEGTLLS	ECGASVKENEGTLLS	0,4353
**chr2:g.166305835G>A**	VGEFTFLRDPWNWLD	VGEFTFLCDPWNWLD	0,4352
**chr1:g.169542001C>T**	SQFLIKTRKKKKEKH	SQFLIKTQKKKKEKH	0,435
**chr20:g.32995693C>T**	KVRHHSGEIQDLRGS	KVRHHSGKIQDLRGS	0,4347
**chr4:g.185623379C>T**	KHERARHENTEEPRR	KHERARHKNTEEPRR	0,4345
**chr7:g.100752054C>T**	PSEKPTISTEKPTVP	PSEKPTIFTEKPTVP	0,4344
**chr5:g.26881510C>T**	YNDEGGGEEDTQAFD	YNDEGGGKEDTQAFD	0,4343
**chr6:g.152416597G>A**	GSSEQRTSCRATADQ	GSSEQRTFCRATADQ	0,4341
**chr8:g.39637607C>T**	WSNENQISTSGDADD	WSNENQIFTSGDADD	0,434
**chr19:g.21971379G>A**	LRYHKKIHTGEKPYK	LRYHKKIYTGEKPYK	0,4338
**chr2:g.124914224C>T**	YPENLDSSFRNEIDL	YPENLDSFFRNEIDL	0,4336
**chr8:g.120283730G>A**	QEPFALWEILNKNSD	QEPFALWKILNKNSD	0,4335
**chr7:g.11593356C>T**	AGTRVRTRTIRQFPI	AGTRVRTQTIRQFPI	0,4334
**chr8:g.53234962G>A**	VLCKIVISIDYYNMF	VLCKIVIFIDYYNMF	0,4333
**chr14:g.99257531C>T**	GIQVTPDEDDHLLSP	GIQVTPDKDDHLLSP	0,4333
**chr12:g.39607657C>T**	TEAFTTARNLLASGA	TEAFTTAQNLLASGA	0,4332
**chr1:g.179618645G>A**	IAQIGPGGTISSELK	IAQIGPGETISSELK	0,433
**chr3:g.99849049C>T**	KEGHLQNGKMQTKPN	KEGHLQNEKMQTKPN	0,4329
**chr4:g.41263246C>T**	VDDKVNFHFILFNNV	VDDKVNFYFILFNNV	0,4329
**chr7:g.93132805G>A**	DETDTLFSPLMEALQ	DETDTLFFPLMEALQ	0,4329
**chr20:g.42678049C>T**	DGPIILKEVEYRTTT	DGPIILKKVEYRTTT	0,4328
**chr7:g.143052920G>A**	CSDTRLLEFWDFLMA	CSDTRLLKFWDFLMA	0,4324
**chr1:g.197008576G>A**	KLYAKTGDAVEFQCK	KLYAKTGNAVEFQCK	0,4322
**chr3:g.96987964G>A**	GRCICSTGYEEIEGS	GRCICSTEYEEIEGS	0,4317
**chr3:g.38847179C>T**	DIFYEVWEKFDPEAT	DIFYEVWKKFDPEAT	0,4316
**chr1:g.247876011G>A**	DVPNNPERFDTWPCI	DVPNNPEQFDTWPCI	0,4308
**chr15:g.24678527C>T**	DRRPTTTSSHPLNTG	DRRPTTTFSHPLNTG	0,4308
**chr1:g.181796713G>A**	SGGRERGRSKERKHL	SGGRERGQSKERKHL	0,4307
**chr1:g.179491561G>A**	MDELHISMIQWMVNL	MDELHISIIQWMVNL	0,4307
**chr2:g.178746747C>T**	VPGVKWYRNKSLLEP	VPGVKWYQNKSLLEP	0,4304
**chr7:g.140753334T>C**	DFGLATVKSRWSGSH	DFGLATVESRWSGSH	0,4304
**chr20:g.31259487G>A**	WIRRCYYGTGRCRKS	WIRRCYYETGRCRKS	0,4303
**chr19:g.37738680G>A**	LTQHQKIHTGGKPYE	LTQHQKIYTGGKPYE	0,43
**chr2:g.130762122C>T**	IKSSLQVSHEKPPDP	IKSSLQVFHEKPPDP	0,43
**chr15:g.43456591G>A**	GSEVEEIPETPCESQ	GSEVEEISETPCESQ	0,43
**chr1:g.171110858C>T**	RVWDNGYPWDMLLVT	RVWDNGYSWDMLLVT	0,4296

**chr4:g.188091461G>A**	GALRPVFSLCIPNGD	GALRPVFFLCIPNGD	0,4296
**chr12:g.123875406C>T**	EVDPRFISLFSVFNV	EVDPRFILLFSVFNV	0,4294
**chr2:g.195787132G>A**	LYGLCFFHALVQERR	LYGLCFFYALVQERR	0,4294
**chr2:g.229025660G>A**	NSSSEEVSQELESDD	NSSSEEVFQELESDD	0,4294
**chr7:g.142252327C>T**	ICNGMLQGILSFADG	ICNGMLQEILSFADG	0,4292
**chr2:g.188994778G>A**	KGEDGKDGSPGEPGA	KGEDGKDRSPGEPGA	0,4289
**chr20:g.31783880C>T**	EDEHFEFHSRPCPTK	EDEHFEFYSRPCPTK	0,4289
**chr6:g.93410659G>A**	TVTGSEFSSLVEVRG	TVTGSEFFSLVEVRG	0,4289
**chr19:g.22391785G>A**	LSKHKVIHTGEKPYK	LSKHKVIYTGEKPYK	0,4288
**chr14:g.71723862C>T**	KPEGTINSVGFMDTR	KPEGTINFVGFMDTR	0,4285
**chr9:g.8524996C>T**	QSEESDQGKYECVAT	QSEESDQEKYECVAT	0,4283
**chr2:g.228017442C>T**	RFMVNQMENEGRGFE	RFMVNQMKNEGRGFE	0,4282
**chr1:g.172032443G>A**	ITKLDLMDEGTDARD	ITKLDLMNEGTDARD	0,4281
**chr1:g.116754910G>A**	VSIYDTKGKNVLEKI	VSIYDTKEKNVLEKI	0,4277
**chr13:g.91693375C>T**	RFFDSLFPLVYNHLI	RFFDSLFSLVYNHLI	0,4276
**chr3:g.169116616G>A**	QMFSTTSSLNKHRRF	QMFSTTSFLNKHRRF	0,4274
**chr19:g.21973311G>A**	LSYHKKIHTVEKPYK	LSYHKKIYTVEKPYK	0,4272
**chr5:g.26881260G>A**	GNDSIADSLSSLESL	GNDSIADLLSSLESL	0,4271
**chr19:g.57129547G>A**	QQGFPNLGNTCYMNA	QQGFPNLENTCYMNA	0,427
**chr1:g.159713724G>A**	ILGQEQDSFGGNFEG	ILGQEQDFFGGNFEG	0,4265
**chr6:g.84055419C>T**	YYEIGPVSFEGLKAH	YYEIGPVFFEGLKAH	0,4263
**chr6:g.10874815G>A**	IPGQPCPSFPKSDVY	IPGQPCPFFPKSDVY	0,4263
**chr3:g.108353712G>A**	RVSLLDEGIYTCYVG	RVSLLDEEIYTCYVG	0,4263
**chr8:g.138141233C>T**	SLSGPHLGTLYNNST	SLSGPHLETLYNNST	0,4261
**chr1:g.56767888G>A**	HNYEATISYLRHSGN	HNYEATILYLRHSGN	0,4255
**chr6:g.93258242C>T**	SGRLKLPGKRDVAVA	SGRLKLPEKRDVAVA	0,4251
**chr3:g.26709859C>T**	KEIPRDLPPETVLLY	KEIPRDLSPETVLLY	0,425
**chr11:g.56376321G>A**	LRMHSAEGRQKAFST	LRMHSAEERQKAFST	0,425
**chr19:g.21973863G>A**	LSYHKKIHTGEKPYK	LSYHKKIYTGEKPYK	0,4247
**chr17:g.42405255G>A**	DAAALELSSDEAVEV	DAAALELLSDEAVEV	0,4244
**chr13:g.24707050G>A**	VVAVTGDGVNDSPAL	VVAVTGDRVNDSPAL	0,4242
**chr8:g.39609521C>T**	QGYAAEFPNSFVTLS	QGYAAEFSNSFVTLS	0,424
**chr2:g.137170884G>A**	GICFPDHGKCGLGHR	GICFPDHRKCGLGHR	0,4234
**chr12:g.21801129C>T**	LVIVMKRGNILEYDT	LVIVMKRENILEYDT	0,4229
**chr4:g.44175160G>A**	KGSESGTSCNELSTS	KGSESGTFCNELSTS	0,4225
**chr7:g.14338598C>T**	WGESKKRRSHRRIEK	WGESKKRQSHRRIEK	0,4224
**chr7:g.142008671G>A**	PVVNELERINCIPDQ	PVVNELEQINCIPDQ	0,4224
**chr22:g.41745929C>T**	EPSAQENPFTAPSAK	EPSAQENLFTAPSAK	0,4215
**chr20:g.44159844C>T**	RSSGLRYEGEWLDNL	RSSGLRYKGEWLDNL	0,4215
**chr2:g.1923177C>T**	NDEYDNYDELVAKSL	NDEYDNYNELVAKSL	0,4215
**chr3:g.52364929C>T**	GIVSDLFPTIKEEDT	GIVSDLFSTIKEEDT	0,4214
**chr2:g.154242088C>T**	TSVVIVFHNEAWSTL	TSVVIVFYNEAWSTL	0,4205
**chr7:g.77262219G>A**	REKERDFRNLRKMEL	REKERDFQNLRKMEL	0,4204

**chr12:g.100380865C>T**	SGLGFCISFGIRCNL	SGLGFCIFFGIRCNL	0,4201
**chr16:g.9764345G>A**	DISETSNRATCHREP	DISETSNWATCHREP	0,4201
**chr6:g.76034649G>A**	IGKNFSNSQEHLDLL	IGKNFSNFQEHLDLL	0,4201
**chr16:g.9764087C>T**	DFPDPYQDPSENFRK	DFPDPYQNPSENFRK	0,42
**chr18:g.53157422G>A**	VPVLVSSRFVRLSWR	VPVLVSSQFVRLSWR	0,4197
**chr7:g.147395735G>A**	EVAQRKPGSFANVSI	EVAQRKPESFANVSI	0,4194
**chr1:g.158355405C>T**	FLSFQGISWEPSPGA	FLSFQGIFWEPSPGA	0,4193
**chr4:g.74446740G>A**	YSEEYDNEPQIPGYI	YSEEYDNKPQIPGYI	0,4189
**chr8:g.92005144C>T**	ELLLDVNENGKRRTP	ELLLDVNKNGKRRTP	0,4188
**chr7:g.71335622C>T**	GGLPATLSPAEEEKA	GGLPATLFPAEEEKA	0,4187
**chr4:g.137531580G>A**	DPDVGENSLHTYSLS	DPDVGENFLHTYSLS	0,4186
**chr7:g.24650540G>A**	VIARILHGGMIDRQG	VIARILHEGMIDRQG	0,4184
**chrX:g.151955752G>A**	TTMLSWVSFWIKTES	TTMLSWVFFWIKTES	0,418
**chr1:g.33743384C>T**	PVLGTFSGNQLPSSI	PVLGTFSENQLPSSI	0,4177
**chr5:g.122450721G>A**	KILRQLLGKEISENV	KILRQLLEKEISENV	0,4176
**chr1:g.7663407C>T**	SAKHRIISPKVEPRT	SAKHRIILPKVEPRT	0,4176
**chr8:g.138143076C>T**	ADFDTMTDRLLDEII	ADFDTMTNRLLDEII	0,4176
**chr2:g.170236049C>T**	DARCDVWSLGITAIE	DARCDVWFLGITAIE	0,4174
**chr6:g.55774207C>T**	VKSAGLVGRQGPQSK	VKSAGLVERQGPQSK	0,4174
**chr1:g.26425451G>A**	GSERRPKGKSMQKRR	GSERRPKEKSMQKRR	0,417
**chr16:g.7595570G>A**	GFGFVTFENSADADR	GFGFVTFKNSADADR	0,4169
**chr8:g.64615122C>T**	AMAAVRDEIDRLLQS	AMAAVRDKIDRLLQS	0,4167
**chr3:g.97448658C>T**	PATKYVFHIRVRTAT	PATKYVFYIRVRTAT	0,4166
**chr2:g.140274565G>A**	HYCVNSESCTIGDDG	HYCVNSELCTIGDDG	0,4163
**chr14:g.19781125C>T**	ARPFDSFSLDKVVSV	ARPFDSFFLDKVVSV	0,416
**chr16:g.61653646C>T**	PRFKRLGELYSVGES	PRFKRLGKLYSVGES	0,4158
**chr6:g.41198180C>T**	LKLQDSGRYWCMRNT	LKLQDSGQYWCMRNT	0,4153
**chr1:g.171117206C>T**	PWLFLTDPKLAMEVY	PWLFLTDSKLAMEVY	0,4153
**chr5:g.13776525C>T**	SPVGEKFRNRALKFP	SPVGEKFQNRALKFP	0,4151
**chr2:g.227032243C>T**	VGPPGPVGIPGLKGE	VGPPGPVEIPGLKGE	0,4149
**chrX:g.102717306C>T**	EPLISAFREFEELAK	EPLISAFCEFEELAK	0,4148
**chr10:g.37219097G>A**	KHQYQEKENKYFEDI	KHQYQEKKNKYFEDI	0,4142
**chr3:g.151193655C>T**	LQYATHFPIPLVSHC	LQYATHFSIPLVSHC	0,4138
**chr10:g.24473372C>T**	PPSPSRIPYGGTRSM	PPSPSRISYGGTRSM	0,4138
**chr11:g.63229372G>A**	KCRRFVHPQWKLIHL	KCRRFVHLQWKLIHL	0,4134
**chr18:g.31329992C>T**	NDNPPVFSMATFAGQ	NDNPPVFLMATFAGQ	0,4131
**chr3:g.122550738G>A**	EQNITSISFPALGTG	EQNITSIFFPALGTG	0,4127
**chr2:g.21002691G>A**	NGSEILFSYFQDLVI	NGSEILFFYFQDLVI	0,4126
**chr18:g.31458505G>A**	RISGVGIDQPPFGIF	RISGVGINQPPFGIF	0,4118
**chr19:g.57135184G>A**	LIQHKKIHTGEKPYE	LIQHKKIYTGEKPYE	0,4117
**chr1:g.115072968G>A**	IAFGLWFRFGGAIKE	IAFGLWFWFGGAIKE	0,4115
**chr19:g.49748415C>T**	DSITSLKEKTNRVNQ	DSITSLKKKTNRVNQ	0,4115
**chr6:g.56179820C>T**	YLFAKSSRFLTKIAV	YLFAKSSQFLTKIAV	0,4115

**chr3:g.51333205C>T**	TWRETGISFVTSVTR	TWRETGIFFVTSVTR	0,4113
**chr11:g.5254395G>A**	HGKKVLTSLGDAIKH	HGKKVLTFLGDAIKH	0,4111
**chr6:g.55774138C>T**	KASEVLLRSVRAANK	KASEVLLQSVRAANK	0,411
**chr9:g.119167845C>T**	HTTFISNEIRLDTFF	HTTFISNKIRLDTFF	0,4109
**chr12:g.7379214C>T**	VNVQGAVGILCANGW	VNVQGAVEILCANGW	0,4106
**chr17:g.53823589G>A**	NTRNPNYEIMHMIEE	NTRNPNYKIMHMIEE	0,4104
**chr1:g.46930174C>T**	LVLKSKNGIHLRLRR	LVLKSKNRIHLRLRR	0,4104
**chr19:g.43185064C>T**	PKVSEGKDVLLLVHN	PKVSEGKNVLLLVHN	0,4096
**chr1:g.114713909G>T**	DILDTAGQEEYSAMR	DILDTAGKEEYSAMR	0,4096
**chr12:g.18738675G>A**	VNDHYPKGNCNMLRK	VNDHYPKENCNMLRK	0,4095
**chr3:g.124446193C>T**	ELLQRENRVLHFWTL	ELLQRENCVLHFWTL	0,4095
**chr12:g.21304474G>A**	PILPLGISYIEDFAK	PILPLGIFYIEDFAK	0,4091
**chr1:g.47116677G>A**	NPLRFSRENSEKIHP	NPLRFSRKNSEKIHP	0,4088
**chr11:g.10487302G>A**	SLHEMLNEMSEFKEL	SLHEMLNKMSEFKEL	0,4087
**chr2:g.188984919C>T**	DCPNPEIPFGECCAV	DCPNPEILFGECCAV	0,4087
**chr7:g.142062634G>A**	TAKWWKREIEELYNN	TAKWWKRKIEELYNN	0,4085
**chr1:g.237784121G>A**	QPFLGRIEIMGSAKR	QPFLGRIKIMGSAKR	0,4085
**chr3:g.17011102G>A**	VEGDVTDEDEGAEMS	VEGDVTDKDEGAEMS	0,4085
**chr4:g.166003528G>A**	HEHTRPDRDNHVTII	HEHTRPDQDNHVTII	0,4085
**chr3:g.12604200G>A**	SLSQRQRSTSTPNVH	SLSQRQRLTSTPNVH	0,4084
**chr12:g.110915742C>T**	DGFIDKNDLRDTFAA	DGFIDKNNLRDTFAA	0,4083
**chr1:g.103754752C>T**	VGFMLAHPYGFTRVM	VGFMLAHSYGFTRVM	0,4082
**chr3:g.17185183G>A**	EELEAQISFLQGQLN	EELEAQIFFLQGQLN	0,4081
**chr17:g.11768516C>T**	EFKTVKFPSQGTIFD	EFKTVKFSSQGTIFD	0,4078
**chr10:g.87894057C>T**	NIIAMGFPAERLEGV	NIIAMGFSAERLEGV	0,4077
**chr9:g.103005172G>A**	SATESEDEKGGAKKD	SATESEDKKGGAKKD	0,4075
**chr14:g.96264209G>A**	SIPTFLLRSIQAVPD	SIPTFLLQSIQAVPD	0,4074
**chr7:g.71670006G>A**	DIGDVSERRALRKSL	DIGDVSEKRALRKSL	0,4073
**chr7:g.93132622G>A**	KMKAPKNSYISDTLG	KMKAPKNFYISDTLG	0,4071
**chr8:g.138151257G>A**	ITHQPLGSFGVVSTH	ITHQPLGFFGVVSTH	0,4069
**chr1:g.248099872C>T**	TVFALHIPYCRSRAI	TVFALHILYCRSRAI	0,4067
**chr5:g.41313697C>T**	GINIVTADFVELGDF	GINIVTANFVELGDF	0,4066
**chr10:g.122640257C>T**	GNFDVNISFYTSSSF	GNFDVNIFFYTSSSF	0,4065
**chrX:g.152189890G>A**	LSISARNSLPKVAYA	LSISARNFLPKVAYA	0,4064
**chr11:g.56031265C>T**	VFYTVVFPMFNPIIY	VFYTVVFSMFNPIIY	0,4063
**chr6:g.62047937C>T**	NSLKRLQEETGAKMS	NSLKRLQKETGAKMS	0,4063
**chr14:g.72474659G>A**	NTTEMDIRKCRRLKN	NTTEMDIQKCRRLKN	0,4062
**chr9:g.990476G>A**	SSVTGAERTSAEPES	SSVTGAEQTSAEPES	0,4062
**chr2:g.140850146C>T**	IKTQTIKRAFINGTG	IKTQTIKQAFINGTG	0,4061
**chr3:g.98264519C>T**	HIPMYLFLGSLAFVD	HIPMYLFFGSLAFVD	0,4059
**chr5:g.13753438G>A**	EMKARKIPFGKNLNL	EMKARKILFGKNLNL	0,4057
**chr3:g.189894387G>A**	ERVIDAVRFTLRQTI	ERVIDAVQFTLRQTI	0,4055
**chr12:g.106239568G>A**	HVEDGVLSMQVASAR	HVEDGVLFMQVASAR	0,4054

**chr8:g.24476473G>A**	PHNKLRNRIWGMVNF	PHNKLRNQIWGMVNF	0,4053
**chr19:g.56003835C>T**	GDKSLTFSSYGLQWC	GDKSLTFFSYGLQWC	0,4052
**chr21:g.40042602C>T**	EELARAYEHAKMEEQ	EELARAYKHAKMEEQ	0,4051
**chr20:g.42098471C>T**	DFWRLVFDYNCSSVV	DFWRLVFNYNCSSVV	0,4048
**chr12:g.7369446C>T**	LDDMRCKGNESFLWD	LDDMRCKENESFLWD	0,4046
**chr5:g.136357258C>T**	GTSLTPEEERFLDSA	GTSLTPEKERFLDSA	0,4045
**chr2:g.167244724C>T**	ESLYEKVSLMTSSEE	ESLYEKVLLMTSSEE	0,4043
**chr20:g.8658677G>A**	QVLIEKYEPNNSLAR	QVLIEKYKPNNSLAR	0,404
**chr5:g.140802174G>A**	QVEATDKGNPPMSDH	QVEATDKENPPMSDH	0,4039
**chr7:g.121271405G>A**	RYKEFLQGKCGCHFH	RYKEFLQEKCGCHFH	0,4033
**chr7:g.142031746G>A**	CAVWWTKEFELFHNQ	CAVWWTKKFELFHNQ	0,4029
**chr21:g.44612219C>T**	PSTCTGSSWQVDNCQ	PSTCTGSFWQVDNCQ	0,4026
**chr11:g.5199868G>A**	HRFGKHLSPVAHVLI	HRFGKHLFPVAHVLI	0,4024
**chr14:g.22924345G>A**	RGPLVNASLRAAKQA	RGPLVNAFLRAAKQA	0,4023
**chr17:g.10496462C>T**	EVDRKIAEKDEEIDQ	EVDRKIAKKDEEIDQ	0,4021
**chr3:g.124268845G>A**	YNHEEWIELRLSLEE	YNHEEWIKLRLSLEE	0,4018
**chr8:g.3409579G>A**	EKGGCGDPGIPAYGK	EKGGCGDSGIPAYGK	0,4016
**chr15:g.51488030G>A**	AALKNAFSLLGKQRF	AALKNAFFLLGKQRF	0,4015
**chr7:g.117559555C>T**	ISFCSQFSWIMPGTI	ISFCSQFFWIMPGTI	0,4013
**chr12:g.32878970C>T**	NLHLVENDFVGGRSP	NLHLVENNFVGGRSP	0,4012
**chr22:g.26311908G>A**	EQGPAIIECINVRDP	EQGPAIIKCINVRDP	0,401
**chr12:g.3278506C>T**	TFSPSFPSLSAANLV	TFSPSFPLLSAANLV	0,4009
**chr3:g.189869372G>A**	QMTSIKKRRSPDDEL	QMTSIKKQRSPDDEL	0,4009
**chr3:g.35792411G>A**	PVLSGQQGFQGLIGV	PVLSGQQRFQGLIGV	0,4007
**chr9:g.21971018G>A**	RDAWGRLPVDLAEEL	RDAWGRLLVDLAEEL	0,4004
**chr7:g.142036869C>T**	ADSLLLNSSRHYLNI	ADSLLLNFSRHYLNI	0,4003
**chr11:g.123939894C>T**	LSGNPTISFGGCLTQ	LSGNPTIFFGGCLTQ	0,4002
**chr1:g.152314540C>T**	KHEDNKQEENKENRK	KHEDNKQKENKENRK	0,4002
**chr8:g.138139037C>T**	LLQLTFRDNADLRKC	LLQLTFRNNADLRKC	0,4001
**chr6:g.110442297G>A**	SLGFYSFSLNSVNLG	SLGFYSFFLNSVNLG	0,4001
**chr3:g.19882615G>A**	SAYSLLYPTIFARKG	SAYSLLYSTIFARKG	0,3999
**chr4:g.185623394C>T**	FTTMLKHERARHENT	FTTMLKHKRARHENT	0,3995
**chr11:g.56989524G>A**	MLLVVFVGSNLIFTG	MLLVVFVESNLIFTG	0,3994
**chr2:g.140322007C>T**	ATCVCPEGKYLINGT	ATCVCPEEKYLINGT	0,3992
**chr5:g.90829077C>T**	SQSLVYFSVGSRLAV	SQSLVYFFVGSRLAV	0,399
**chr4:g.55090053C>T**	IHRDLAARNILLSEK	IHRDLAAQNILLSEK	0,3987
**chr10:g.123780226C>T**	NYYHRRNEMTTTDDL	NYYHRRNKMTTTDDL	0,398
**chr10:g.53827519C>T**	AECTKTARIQAALPA	AECTKTAQIQAALPA	0,3976
**chr7:g.53036234G>A**	ALSQCPKGSARFDGP	ALSQCPKESARFDGP	0,3971
**chr19:g.54255310C>T**	PRWSAPSDPLDILIA	PRWSAPSNPLDILIA	0,3968
**chr1:g.197435318G>A**	SNPCLHGGNCEDIYS	SNPCLHGENCEDIYS	0,3968
**chr1:g.40546948C>T**	LATHQKIHNGERPFV	LATHQKIYNGERPFV	0,3967
**chr1:g.181776188G>A**	LGPHHLDEFVRVWAE	LGPHHLDKFVRVWAE	0,3966

**chr20:g.9566078G>A**	RVSHEQFRAALQLVV	RVSHEQFWAALQLVV	0,3964
**chr19:g.43258283G>A**	IPQITRNHSGLYACS	IPQITRNYSGLYACS	0,3964
**chr12:g.70552832C>T**	ESLGGKCDPTQQKFC	ESLGGKCNPTQQKFC	0,3963
**chr12:g.52451682C>T**	GFPVCPPGGIQEVTV	GFPVCPPRGIQEVTV	0,3963
**chr8:g.68217680C>T**	RSHGLPPRYIMQATD	RSHGLPPCYIMQATD	0,3963
**chr8:g.2002532C>T**	PLPRGAFAVAHEATT	PLPRGAFVVAHEATT	0,3963
**chr11:g.48245686G>A**	GLLIVANGGTLSVIS	GLLIVANRGTLSVIS	0,396
**chr6:g.54940959G>A**	TENLLKRRSFPLFDN	TENLLKRQSFPLFDN	0,3956
**chr7:g.82952858G>A**	SSISITIPPEPLALD	SSISITISPEPLALD	0,3953
**chr20:g.9566119G>A**	PPPSWGSSSDQQPSR	PPPSWGSFSDQQPSR	0,395
**chr14:g.73253816C>T**	GGGSQSRSVYCISSD	GGGSQSRFVYCISSD	0,3948
**chr21:g.14165304G>A**	NCNFISFPCRSYKDY	NCNFISFSCRSYKDY	0,3947
**chrX:g.136346323G>A**	TMLEVTDESAQRVTA	TMLEVTDKSAQRVTA	0,3945
**chr20:g.18526475C>T**	DELKIPIRSWHDIEK	DELKIPICSWHDIEK	0,3942
**chr20:g.6112599G>A**	IRRSEELSLLKPSGD	IRRSEELFLLKPSGD	0,3941
**chr12:g.11267992C>T**	EGPPPQGGNQSQGPP	EGPPPQGENQSQGPP	0,394
**chr4:g.154320457C>T**	ITAHDPDEGRNGKVT	ITAHDPDKGRNGKVT	0,394
**chr19:g.44429696G>A**	GKGFRYKSVLLIHQG	GKGFRYKLVLLIHQG	0,394
**chr7:g.34153166G>A**	KTCDNWNEIGPCNKP	KTCDNWNKIGPCNKP	0,3935
**chr12:g.79296132C>T**	DMGGTSDPYVKVFLL	DMGGTSDSYVKVFLL	0,3935
**chr7:g.77199535C>T**	KEVQNLKEIVNSLKK	KEVQNLKKIVNSLKK	0,3933
**chr12:g.45836858C>T**	AVILRNLSFEEGNVK	AVILRNLFFEEGNVK	0,3933
**chr1:g.192184490G>A**	RPTNLRRRSRSFTCN	RPTNLRRQSRSFTCN	0,393
**chr7:g.98912179C>T**	KLVFGSVSLFAAENE	KLVFGSVFLFAAENE	0,3929
**chr10:g.26128459C>T**	FENFKKNSFEQLCIN	FENFKKNFFEQLCIN	0,3927
**chr8:g.3586237G>A**	VGANVQFSCEDNYVL	VGANVQFLCEDNYVL	0,3927
**chr17:g.60057151G>A**	WLIRLCISIVVLPKE	WLIRLCIFIVVLPKE	0,3926
**chr8:g.138826705C>T**	FRKTSRKEDWYIWQV	FRKTSRKKDWYIWQV	0,3925
**chr7:g.18648517C>T**	LATKERISPGIRGTH	LATKERILPGIRGTH	0,3924
**chr15:g.66436825C>T**	VLHECNSPYIVGFYG	VLHECNSLYIVGFYG	0,3923
**chr3:g.96987681C>T**	LKLNTEIREVGPIER	LKLNTEICEVGPIER	0,3922
**chrX:g.34131147C>T**	RGSSLHAEPSKTGVS	RGSSLHAKPSKTGVS	0,3919
**chr11:g.100255795G>A**	PGIVIVEEITESTAT	PGIVIVEKITESTAT	0,3917
**chr5:g.11111006G>A**	VCILRNLSYRLAAET	VCILRNLLYRLAAET	0,3916
**chr1:g.158399186G>A**	LSDTKTISFMACATQ	LSDTKTIFFMACATQ	0,3911
**chr9:g.101686843G>A**	GVMPPELRWVLGDSQ	GVMPPELCWVLGDSQ	0,3911
**chr12:g.113099994G>A**	EVLADLDRAHEEFQQ	EVLADLDCAHEEFQQ	0,3911
**chr1:g.56693791G>A**	SNMMSDGEFLRTSCG	SNMMSDGKFLRTSCG	0,3908
**chr20:g.42677928C>T**	EIRVLLTRPGEGGTG	EIRVLLTQPGEGGTG	0,3906
**chr1:g.37761861C>T**	TFNVYYLETEADLGR	TFNVYYLKTEADLGR	0,3904
**chr11:g.55968291G>A**	YFFLSNFSLLEICYV	YFFLSNFFLLEICYV	0,3901
**chr2:g.137657119G>A**	SLWNNNERTVWCQRS	SLWNNNEQTVWCQRS	0,3899
**chr14:g.91669903G>A**	PNSQSKFPIFRFPSS	PNSQSKFSIFRFPSS	0,3895

**chr9:g.21974685G>A**	PNSYGRRPIQVGRGS	PNSYGRRLIQVGRGS	0,3895
**chr6:g.83857331C>T**	FPIQATISFYEDSDS	FPIQATILFYEDSDS	0,3891
**chr1:g.213997055C>T**	ARVENIIRGMSHSPS	ARVENIIWGMSHSPS	0,3889
**chr1:g.223003063C>T**	VFHFEFFPFMNLTAL	VFHFEFFSFMNLTAL	0,3888
**chr2:g.228017733C>T**	SVNLFANEVAAKIMN	SVNLFANKVAAKIMN	0,3886
**chr7:g.82915855C>T**	DIDHHTPRNYVLIDD	DIDHHTPQNYVLIDD	0,3882
**chr12:g.119505202C>T**	MISVNPGSDEPPSVN	MISVNPGLDEPPSVN	0,3879
**chr2:g.228017993C>T**	ICLDNSSGKQPWFCA	ICLDNSSEKQPWFCA	0,3878
**chr13:g.24825661G>A**	FLLKTIEEFYKSEDG	FLLKTIEKFYKSEDG	0,3876
**chr18:g.63804321C>T**	KYVEVFFPQFKIEKN	KYVEVFFSQFKIEKN	0,3871
**chr7:g.77241866G>A**	NEYNAVKEREFHNQY	NEYNAVKKREFHNQY	0,387
**chr14:g.56801997G>A**	FGGMDCGSYLTPMHH	FGGMDCGLYLTPMHH	0,3864
**chr8:g.3359247G>A**	VGDSLTFSCFLGYRL	VGDSLTFFCFLGYRL	0,3858
**chr2:g.214014012C>T**	VNSIEFFPFSNTLLT	VNSIEFFSFSNTLLT	0,3857
**chr3:g.38605998C>T**	IAETEEKEKRFQEAM	IAETEEKKKRFQEAM	0,3856
**chr1:g.240206904C>T**	AELERQYPALDTEVA	AELERQYSALDTEVA	0,3854
**chr2:g.112019498G>A**	SNRNFLHRDLAARNC	SNRNFLHQDLAARNC	0,3854
**chr19:g.9467172C>T**	SYLTKHLRRHSGEKP	SYLTKHLQRHSGEKP	0,3853
**chr6:g.130441582C>T**	RQFGSNTSLHLLSSH	RQFGSNTFLHLLSSH	0,3852
**chr14:g.21325861G>A**	ESWEPQNELWIEITK	ESWEPQNKLWIEITK	0,3851
**chr5:g.32091075G>A**	GNRACPGGSGPKTSA	GNRACPGRSGPKTSA	0,385
**chr16:g.71184966C>T**	TPSEFLKEMSLTTEQ	TPSEFLKKMSLTTEQ	0,3847
**chr10:g.71288667G>A**	MAPPPQNGGRDCSGT	MAPPPQNEGRDCSGT	0,3847
**chr8:g.3230208C>T**	SIAATCNDPGMPQNG	SIAATCNNPGMPQNG	0,3846
**chr4:g.157136613G>A**	PDLFFANEKSANFHD	PDLFFANKKSANFHD	0,3841
**chr20:g.44054333G>A**	KRPSADPGKKAKNPK	KRPSADPEKKAKNPK	0,3839
**chr1:g.97740427C>T**	KSCPTNLDIKSFITS	KSCPTNLNIKSFITS	0,3838
**chr16:g.78030575G>A**	VIPRNSDEINALQDY	VIPRNSDKINALQDY	0,3837
**chr1:g.94188873C>T**	ESTGGSSESRSLDSE	ESTGGSSKSRSLDSE	0,3837
**chr14:g.19828286C>T**	LFHTVIFPLLNPVIY	LFHTVIFSLLNPVIY	0,3837
**chr17:g.10455041C>T**	GLLGTLEEMRDEKLA	GLLGTLEKMRDEKLA	0,3832
**chrX:g.111912725C>T**	AAHTNNYEIIKLLVQ	AAHTNNYKIIKLLVQ	0,3826
**chr10:g.27995079C>T**	GHGTGILEITPLNEA	GHGTGILKITPLNEA	0,3826
**chr1:g.206116373G>A**	LPQVFIFSLREVIQG	LPQVFIFFLREVIQG	0,3822
**chr12:g.111308493C>T**	FFPTQKFLLEKPSLL	FFPTQKFFLEKPSLL	0,3819
**chr16:g.53645651G>A**	KVNVPLISLAHDRCI	KVNVPLILLAHDRCI	0,3817
**chr3:g.131685919G>A**	DPFLEIFRMNDDATQ	DPFLEIFCMNDDATQ	0,3817
**chr7:g.88131435C>T**	SDAVHLFSYVTSVMM	SDAVHLFLYVTSVMM	0,3816
**chr16:g.84486777G>A**	HRWCLLFSSELHGHS	HRWCLLFLSELHGHS	0,3815
**chr4:g.20732755C>T**	DIMKAIYDMMGKCTY	DIMKAIYNMMGKCTY	0,3813
**chr14:g.88740535C>T**	GNALTPKRGVFGKTG	GNALTPKQGVFGKTG	0,3808
**chr6:g.76034743G>A**	AYRIFLDRIPDTGEY	AYRIFLDCIPDTGEY	0,3807
**chr1:g.81943106C>T**	FMPWTPYRTDTLIEY	FMPWTPYCTDTLIEY	0,3796

**chr1:g.47099244G>A**	HQQRCRDEIRELLGD	HQQRCRDKIRELLGD	0,3795
**chr2:g.108010263G>A**	KEIVWVMRITVFVFG	KEIVWVMQITVFVFG	0,3794
**chr11:g.56030590C>T**	INSSLQIPMYYFLSN	INSSLQISMYYFLSN	0,3793
**chr2:g.186839020C>T**	QKEIDSEDICSICQE	QKEIDSENICSICQE	0,3792
**chr12:g.43432345C>T**	VTRENCNEFSCPSWA	VTRENCNKFSCPSWA	0,3779
**chr4:g.157136847C>T**	PLDLTLFPMDTQRCK	PLDLTLFSMDTQRCK	0,3773
**chr17:g.12752558C>T**	DITTVTFPVTPNTLP	DITTVTFSVTPNTLP	0,3772
**chr7:g.81752150C>T**	SNPEVRYEVCDIPQC	SNPEVRYKVCDIPQC	0,3771
**chrX:g.105767263G>A**	FWKHLVYEMPIKKKE	FWKHLVYKMPIKKKE	0,3769
**chr5:g.147661112C>T**	ERLKLLQEIADLKTA	ERLKLLQKIADLKTA	0,3769
**chr17:g.76018975C>T**	DVAPLPQRRNPPQQP	DVAPLPQQRNPPQQP	0,3768
**chr11:g.56105320C>T**	LLTSNYISFTGCFAQ	LLTSNYIFFTGCFAQ	0,3768
**chr16:g.51140199G>A**	EQFKAKFPFGGLLDS	EQFKAKFSFGGLLDS	0,3767
**chr1:g.171208906C>T**	CSLLFKDPKLAVRLY	CSLLFKDSKLAVRLY	0,3765
**chr2:g.30751126G>A**	KAEDAKFPLDFQVIL	KAEDAKFSLDFQVIL	0,3764
**chr17:g.63880884G>A**	LIQSWLEPVQLLRSV	LIQSWLELVQLLRSV	0,3763
**chr2:g.48669854G>A**	TRDADENEFLGNIDG	TRDADENKFLGNIDG	0,376
**chr3:g.36857219C>T**	DFDNMTWEIECTSEM	DFDNMTWKIECTSEM	0,3759
**chr10:g.105158934C>T**	HLQLSENPYSSGRIS	HLQLSENSYSSGRIS	0,3759
**chr7:g.142065390C>T**	VITRSTFPSSGRWAG	VITRSTFSSSGRWAG	0,3758
**chr6:g.32070316G>A**	LRLSWTVPEGQFDSF	LRLSWTVSEGQFDSF	0,3757
**chr10:g.1217054C>T**	RTKIESGEGTVPVRG	RTKIESGKGTVPVRG	0,3757
**chr8:g.124082425G>A**	NRFYSKHRAICGLQS	NRFYSKHQAICGLQS	0,3757
**chr4:g.106924058C>T**	KKGSHGLEIFQRCDC	KKGSHGLKIFQRCDC	0,3746
**chr2:g.182240168C>T**	MTKKKPEEKPKFRSI	MTKKKPEKKPKFRSI	0,3744
**chr6:g.29440793C>T**	TALFIYIRPKASYDP	TALFIYICPKASYDP	0,3744
**chr12:g.57751653T>A**	GAYGTVYKARDPHSG	GAYGTVYMARDPHSG	0,3743
**chr3:g.38894601C>T**	LAPLAEEEDDVEFSG	LAPLAEEKDDVEFSG	0,3743
**chr10:g.37217759G>A**	ILNEKIREELGRIEE	ILNEKIRKELGRIEE	0,3743
**chr1:g.97699420G>A**	PASISCASFLARLGY	PASISCAFFLARLGY	0,3741
**chr11:g.7960552G>A**	LYKACQVPGICWVVC	LYKACQVSGICWVVC	0,3741
**chr19:g.53634561G>A**	QMHSHRKRTMFTKKQ	QMHSHRKQTMFTKKQ	0,3738
**chr5:g.42718080C>T**	MLILPPVPVPKIKGI	MLILPPVSVPKIKGI	0,3737
**chr11:g.56361096C>T**	VFYTLVIPMLNPLIY	VFYTLVISMLNPLIY	0,3736
**chr8:g.132140119C>T**	YGNDFPIEDMIPTLK	YGNDFPIKDMIPTLK	0,3734
**chr18:g.31392189C>T**	IEENCLSSELIRLQA	IEENCLSLELIRLQA	0,3734
**chr10:g.122598872G>A**	GSWGTVCDDSWDTND	GSWGTVCNDSWDTND	0,3732
**chr4:g.46064480G>A**	YLQLHNFPMDEHSCP	YLQLHNFSMDEHSCP	0,373
**chr12:g.18738513G>A**	HNVMGDYRFFDHQSK	HNVMGDYQFFDHQSK	0,373
**chr2:g.40428861C>T**	KPGDTQKEIRVGIID	KPGDTQKKIRVGIID	0,373
**chr12:g.41572467G>A**	GRTDESLRNDESSEQ	GRTDESLQNDESSEQ	0,3727
**chr11:g.5488993C>T**	WLHAGEISFGGCLAQ	WLHAGEIFFGGCLAQ	0,3721
**chrX:g.70204342G>A**	SYSFGENEVFNQETF	SYSFGENKVFNQETF	0,372

**chr14:g.19936109C>T**	CVIFVSISWAVGVLH	CVIFVSIFWAVGVLH	0,372
**chr8:g.138265726G>A**	DAVVFRVHLLLGGER	DAVVFRVYLLLGGER	0,372
**chr11:g.121558637C>T**	DLKPGIYRSNMDGSA	DLKPGIYWSNMDGSA	0,372
**chr11:g.56290788G>A**	LLTSNYISFMGCFAQ	LLTSNYIFFMGCFAQ	0,3719
**chr16:g.20324229G>A**	GNPIEASSYGLDLDC	GNPIEASLYGLDLDC	0,3719
**chr11:g.4946794C>T**	SNACFAQEFFIHGFS	SNACFAQKFFIHGFS	0,3718
**chrX:g.141241615C>T**	VKRTSPEELVNDHAR	VKRTSPEKLVNDHAR	0,3716
**chr3:g.98470423C>T**	ILFTIVVPLLNPFIY	ILFTIVVSLLNPFIY	0,3711
**chr3:g.36855737C>T**	TFEVFKNEIWPKMTK	TFEVFKNKIWPKMTK	0,3708
**chr11:g.92355186C>T**	PVSLSSVSFVEVEVV	PVSLSSVFFVEVEVV	0,3703
**chr19:g.55818111C>T**	LENLSDKEFQSFKKY	LENLSDKKFQSFKKY	0,3702
**chr12:g.48864849C>T**	TYVPTVFENYTACLE	TYVPTVFKNYTACLE	0,37
**chr20:g.21706617G>A**	KILARYNETGSILPG	KILARYNKTGSILPG	0,3698
**chr19:g.40035544G>A**	LIQHQKIHSNEKPFV	LIQHQKIYSNEKPFV	0,3696
**chr19:g.54888073C>T**	LMPGENISLTCSSAH	LMPGENIFLTCSSAH	0,369
**chr8:g.132184297C>T**	WAAGCCCRYKGWRGR	WAAGCCCQYKGWRGR	0,369
**chrX:g.57378677C>T**	VPNKGQFPLAVGAQE	VPNKGQFSLAVGAQE	0,3689
**chr5:g.161689297C>T**	PMRLVNFPMDGHACP	PMRLVNFSMDGHACP	0,3689
**chr11:g.40115752C>T**	LRRLDLGELKRLSYI	LRRLDLGKLKRLSYI	0,3686
**chr19:g.55732171C>T**	IGLFETQEKEFVTKV	IGLFETQKKEFVTKV	0,3686
**chr3:g.96987936C>T**	ADGDWLVPLGRCICS	ADGDWLVSLGRCICS	0,3685
**chr12:g.5045832C>T**	KVSGSRGSFCKAGGT	KVSGSRGFFCKAGGT	0,3685
**chr5:g.151266845C>T**	DVLDMLIPTGEPCPE	DVLDMLISTGEPCPE	0,3684
**chr2:g.137572473C>T**	LCNQDEIPPETQSCS	LCNQDEISPETQSCS	0,3683
**chr7:g.150692381G>A**	IILVGKTGTGKSAAG	IILVGKTETGKSAAG	0,368
**chr11:g.94104615G>A**	RRSDVDYEFALLFLV	RRSDVDYKFALLFLV	0,3678
**chr17:g.49042324G>A**	NCCRAEQEIMKKVRE	NCCRAEQKIMKKVRE	0,3677
**chr3:g.189886417C>T**	TSIQSPSSYGNSSPP	TSIQSPSLYGNSSPP	0,3676
**chr2:g.60461620G>A**	MKTHMHKSSPMTVKS	MKTHMHKLSPMTVKS	0,3675
**chr7:g.99710758C>T**	VQQKVQKEIDTVLPN	VQQKVQKKIDTVLPN	0,3675
**chr20:g.5302303G>A**	FYGFTIVRDFFPTVF	FYGFTIVCDFFPTVF	0,3674
**chr5:g.41195871C>T**	DCGDNSDERDCGRTK	DCGDNSDKRDCGRTK	0,3673
**chr5:g.168806477G>A**	GKGLMEIPANLPEGI	GKGLMEISANLPEGI	0,3669
**chr6:g.69354300C>T**	INADSSSSFPNGHAQ	INADSSSLFPNGHAQ	0,3668
**chr5:g.42718570G>A**	LDIDEPDEKTEESDT	LDIDEPDKKTEESDT	0,3663
**chr12:g.34026739C>T**	PQKLLEFRYFILPYV	PQKLLEFCYFILPYV	0,3661
**chr6:g.7373393C>T**	ALDLLKREKEAQEQE	ALDLLKRKKEAQEQE	0,3656
**chrX:g.85271394C>T**	SVHTKDFPHKCEVCD	SVHTKDFSHKCEVCD	0,3655
**chr5:g.140968052G>A**	VVSGPLDRERVAVYN	VVSGPLDQERVAVYN	0,3653
**chr11:g.5581583C>T**	DIMLITVSYIHILQA	DIMLITVFYIHILQA	0,3652
**chr1:g.196469026C>T**	LRIPFILEIINAVPF	LRIPFILKIINAVPF	0,3652
**chr7:g.92062348C>T**	EKTDIIDRLEQELLC	EKTDIIDCLEQELLC	0,3651
**chr5:g.13794030C>T**	IESYVDKRMGTTYGP	IESYVDKQMGTTYGP	0,3651

**chr4:g.99313880C>T**	PIQEVLKEMTDGGVD	PIQEVLKKMTDGGVD	0,365
**chr11:g.5544850G>A**	DVILIAVSYAHILCA	DVILIAVFYAHILCA	0,365
**chr19:g.49744265G>A**	DLSMNLDRSHQGNCA	DLSMNLDWSHQGNCA	0,365
**chr2:g.137056548G>A**	ESNRPPKERSCFRVC	ESNRPPKKRSCFRVC	0,3646
**chr5:g.178981730C>T**	PEQNVQKRKRSLKAT	PEQNVQKQKRSLKAT	0,3645
**chr8:g.24395753G>A**	YKGNILNEKNSVASI	YKGNILNKKNSVASI	0,3644
**chr3:g.55734806C>T**	EVMALKREKDRLVHQ	EVMALKRKKDRLVHQ	0,3644
**chr1:g.18482884G>A**	TATVRAKEIFVTGGS	TATVRAKKIFVTGGS	0,3644
**chr17:g.7673781C>T**	RVCACPGRDRRTEEE	RVCACPGKDRRTEEE	0,3643
**chr3:g.172448110G>A**	CMPLDLVRLWQYRPW	CMPLDLVCLWQYRPW	0,3639
**chr6:g.54870398G>A**	QEFLVQERVSDFLAE	QEFLVQEQVSDFLAE	0,3638
**chr12:g.33407312G>A**	LPRQMQVSSVDFSMG	LPRQMQVFSVDFSMG	0,3638
**chrX:g.152135035G>A**	IDVKEVDPTGHSFVL	IDVKEVDSTGHSFVL	0,3636
**chr1:g.114713908T>A**	DILDTAGQEEYSAMR	DILDTAGLEEYSAMR	0,3636
**chr12:g.130445244C>T**	NPFDGPNENPEAELP	NPFDGPNKNPEAELP	0,3635
**chr12:g.101126910C>T**	FCIKHLISYLIPDLP	FCIKHLILYLIPDLP	0,3631
**chr8:g.11550230C>T**	APINKAGSFLIRESE	APINKAGFFLIRESE	0,363
**chr14:g.51966649C>T**	PVPASENPFREKKFF	PVPASENSFREKKFF	0,3627
**chr6:g.38917359G>A**	KIDPVTMDPEKSCCK	KIDPVTMNPEKSCCK	0,3625
**chrX:g.141908073C>T**	DTLLESDSLTDSESL	DTLLESDFLTDSESL	0,3625
**chr1:g.56933382G>A**	EFQKEVSSCHCAPCQ	EFQKEVSFCHCAPCQ	0,3618
**chr11:g.48306763C>T**	NFFCDVHPLLKLACA	NFFCDVHSLLKLACA	0,3616
**chr14:g.19936252G>A**	CMDTYEMEIMTLTNS	CMDTYEMKIMTLTNS	0,3616
**chr5:g.76597570G>A**	AAAMYQNELFNLQKQ	AAAMYQNKLFNLQKQ	0,3614
**chr14:g.62779889C>T**	FGDIFWKETTLAHAC	FGDIFWKKTTLAHAC	0,361
**chr6:g.38883904G>A**	IESYVDKRIGSTYGP	IESYVDKQIGSTYGP	0,3609
**chr3:g.108953648C>T**	SGLQTLHEFKTLLGL	SGLQTLHKFKTLLGL	0,3607
**chr6:g.125076804C>T**	SIFGCKYRYLPERPH	SIFGCKYCYLPERPH	0,3607
**chr19:g.57131247G>A**	QAWFTYNDLCVSEIS	QAWFTYNNLCVSEIS	0,3606
**chr14:g.67203164C>T**	VTVGICFSSSTHKIP	VTVGICFFSSTHKIP	0,3605
**chr12:g.43376112C>T**	MYLENPKEYLTLVQG	MYLENPKKYLTLVQG	0,3604
**chr19:g.45472903C>T**	VNPSYTSSFVLTCPE	VNPSYTSLFVLTCPE	0,3604
**chrX:g.74740623C>T**	LLDDDQREFQEPSYI	LLDDDQRKFQEPSYI	0,3603
**chr3:g.111885242G>A**	NFSCGSVEFDEADLE	NFSCGSVKFDEADLE	0,3603
**chr12:g.70587078C>T**	FTDLVPGRKYMATVT	FTDLVPGQKYMATVT	0,3601
**chr7:g.144050762G>A**	ILVMMSYDRYMAICH	ILVMMSYNRYMAICH	0,36
**chr6:g.127829539G>A**	WDSTNPFPKDFYGTL	WDSTNPFSKDFYGTL	0,3593
**chr5:g.31323099G>A**	FINQRLKENDTDPTA	FINQRLKKNDTDPTA	0,3592
**chr19:g.10020765G>A**	FALEGFFESLAIQLL	FALEGFFKSLAIQLL	0,3585
**chr10:g.122576638G>A**	DVRCSGHESYLWSCP	DVRCSGHKSYLWSCP	0,3581
**chr12:g.110884164G>A**	IEDFLAKEEKNFARF	IEDFLAKKEKNFARF	0,358
**chr5:g.136251859C>T**	MIWSECKEIWEEGPR	MIWSECKKIWEEGPR	0,3579
**chr3:g.121279933G>A**	QRQPRSPRKNKQFIA	QRQPRSPQKNKQFIA	0,3578

**chr11:g.58724447G>A**	MLQMLEKSLRKSLPA	MLQMLEKFLRKSLPA	0,3577
**chr4:g.165995102G>A**	KQAMRHWEKHTCVTF	KQAMRHWKKHTCVTF	0,3576
**chr5:g.122444611G>A**	TMQNHAGEKPSQSAE	TMQNHAGKKPSQSAE	0,3576
**chr11:g.123943189G>A**	SLALTSVSYACILAT	SLALTSVFYACILAT	0,3573
**chr11:g.4914921G>A**	KALNTCVSHICAVLL	KALNTCVFHICAVLL	0,3572
**chr12:g.39720856G>A**	RMTIKTKEKMNNFYV	RMTIKTKKKMNNFYV	0,3568
**chr12:g.20880934G>A**	NCDESQWEPVCGNNG	NCDESQWKPVCGNNG	0,3567
**chr19:g.56028442C>T**	CPYLRKIRVDVKGIF	CPYLRKIWVDVKGIF	0,3567
**chr19:g.51146089C>T**	SLGSQHVSLNLSLQQ	SLGSQHVFLNLSLQQ	0,3567
**chr17:g.7674241G>A**	YNYMCNSSCMGGMNR	YNYMCNSFCMGGMNR	0,3567
**chr16:g.51141362G>A**	NPLSTLSSHLSQQLA	NPLSTLSFHLSQQLA	0,3561
**chr7:g.101042664G>A**	MSVSMPMEISTLGTT	MSVSMPMKISTLGTT	0,3561
**chr11:g.6846184C>T**	TTWLFSFPFCGTNKV	TTWLFSFSFCGTNKV	0,3557
**chr3:g.2866802C>T**	YPSYQDNRRFVSQET	YPSYQDNCRFVSQET	0,3557
**chr7:g.143478446C>T**	KLVIWSIPFSVFLVS	KLVIWSISFSVFLVS	0,3551
**chr8:g.51408344C>T**	SPSRIIKEGGIDPVL	SPSRIIKKGGIDPVL	0,3551
**chr7:g.122698111G>A**	YKPNDIVRILTCKHF	YKPNDIVCILTCKHF	0,3546
**chr18:g.47028921C>T**	SAPALQEEAAFPGRR	SAPALQEKAAFPGRR	0,3544
**chr2:g.195960849G>A**	DGLNPYLRLYETAVE	DGLNPYLCLYETAVE	0,3541
**chr8:g.51426735G>A**	LAVFTQLPQDTSVEV	LAVFTQLSQDTSVEV	0,3541
**chr11:g.5986606C>T**	VLLVHAHEIGYIVCL	VLLVHAHKIGYIVCL	0,3539
**chr22:g.24225581C>T**	ELLDLCERCPRGSGT	ELLDLCEQCPRGSGT	0,3536
**chr7:g.140781608G>A**	GQRIGSGSFGTVYKG	GQRIGSGLFGTVYKG	0,3536
**chr17:g.40798321C>T**	GLRRVLDELTLCRTD	GLRRVLDKLTLCRTD	0,3536
**chr14:g.99878570C>T**	DSRGNRNRTGSTSSS	DSRGNRNCTGSTSSS	0,3531
**chr8:g.16110174C>T**	EVFCFGRESSIEECK	EVFCFGRKSSIEECK	0,3521
**chr2:g.112913064G>A**	KMGSEDWEKDEPQCC	KMGSEDWKKDEPQCC	0,352
**chr8:g.66444682C>T**	PVQVAMDSLVKNGIP	PVQVAMDFLVKNGIP	0,3519
**chr4:g.166025369C>T**	NLSSPGFPNGYPSYT	NLSSPGFSNGYPSYT	0,3517
**chr8:g.66076829G>A**	VAEQFGDENVNTYFM	VAEQFGDKNVNTYFM	0,3516
**chr9:g.134817057C>T**	GPTGEPGPSGPPGKR	GPTGEPGLSGPPGKR	0,3513
**chr19:g.55912571C>T**	LQQLRKNETLFHSCS	LQQLRKNKTLFHSCS	0,3512
**chr11:g.4367640G>A**	DVVLIFISYMLILHA	DVVLIFIFYMLILHA	0,3512
**chr6:g.83398482C>T**	MDLQDRNEKLFYRVL	MDLQDRNKKLFYRVL	0,351
**chr12:g.2595957C>T**	FILLSSISLAAEDPV	FILLSSIFLAAEDPV	0,3508
**chr7:g.140753337C>T**	GDFGLATVKSRWSGS	GDFGLATMKSRWSGS	0,3508
**chr7:g.143052534C>T**	YFFLGNFSLLEILVT	YFFLGNFFLLEILVT	0,3502
**chr7:g.82916495G>A**	LQGVAEDRDYMSDSE	LQGVAEDCDYMSDSE	0,3495
**chr1:g.43403240C>T**	YRISRNVRLAWFLSH	YRISRNVCLAWFLSH	0,3494
**chr9:g.132499569C>T**	KKEPPPLPPNMTFGI	KKEPPPLSPNMTFGI	0,3489
**chr11:g.4821846G>A**	TTILTNARIAKIGMS	TTILTNAQIAKIGMS	0,3488
**chr7:g.88282916G>A**	EKKDNTFRMAISIPN	EKKDNTFCMAISIPN	0,3487
**chr8:g.76852088C>T**	PAFLPHFPMTPEALL	PAFLPHFSMTPEALL	0,3486

**chr1:g.13416463C>T**	ELPTELFPPLFMEAF	ELPTELFSPLFMEAF	0,3485
**chr1:g.7803123C>T**	SYLHPEDRSLMVAIH	SYLHPEDCSLMVAIH	0,3485
**chr3:g.180659703C>T**	KRKILDNELTETISA	KRKILDNKLTETISA	0,3483
**chr6:g.68993796G>A**	EHLAKGQRMLAGDGM	EHLAKGQQMLAGDGM	0,3481
**chr6:g.55359087G>A**	RTITQSEESLCKIFQ	RTITQSEKSLCKIFQ	0,348
**chr5:g.161294262C>T**	LPRHSFGRNALERHV	LPRHSFGQNALERHV	0,3473
**chr8:g.24330030G>A**	VAGTMAHEMGHNFGM	VAGTMAHKMGHNFGM	0,3471
**chr8:g.72567795C>T**	SRSTLSLPPEPVDII	SRSTLSLSPEPVDII	0,3466
**chr1:g.56933392C>T**	ALEEFQKEVSSCHCA	ALEEFQKKVSSCHCA	0,3465
**chr18:g.31459931G>A**	AFKIVSQEPAGTPMF	AFKIVSQKPAGTPMF	0,3465
**chr2:g.100302983C>T**	RSKEVLKEFLYCLAL	RSKEVLKKFLYCLAL	0,3464
**chr1:g.115286402C>T**	HPIFHRGEFSVCDSV	HPIFHRGKFSVCDSV	0,3461
**chr14:g.38254747G>A**	GLVKLCFHESPSSQP	GLVKLCFYESPSSQP	0,3457
**chr5:g.37337847G>A**	CVNHDTFPFQKPMME	CVNHDTFSFQKPMME	0,3456
**chr22:g.35082544C>T**	RKSKRRVRTTFTTEQ	RKSKRRVCTTFTTEQ	0,3456
**chr18:g.61816181G>A**	TPVFIKLPSNGCYML	TPVFIKLSSNGCYML	0,3455
**chr1:g.181717161G>A**	VSYFRHKERLLRISI	VSYFRHKKRLLRISI	0,3455
**chr2:g.233636967C>T**	FDAVFLDPFDTCGLI	FDAVFLDSFDTCGLI	0,3445
**chr9:g.27173335G>A**	YGCSCATGWKGLQCN	YGCSCATSWKGLQCN	0,3444
**chr3:g.108410685C>T**	LLDASQKEVQALSTE	LLDASQKKVQALSTE	0,3442
**chr3:g.54503561G>A**	NFLELGKEFILAPND	NFLELGKKFILAPND	0,3439
**chr21:g.40080182C>T**	VGPGRISEIIEAKTL	VGPGRISKIIEAKTL	0,3438
**chr3:g.17009803C>T**	GSELKKVRSNSRIYH	GSELKKVCSNSRIYH	0,3437
**chrX:g.139956051G>A**	KSSSRSFRKLDSRNN	KSSSRSFCKLDSRNN	0,3437
**chr8:g.100529713G>A**	SGFDPNIRDSRGRTG	SGFDPNICDSRGRTG	0,3429
**chr10:g.122621356G>A**	THNCGHHEDAGVICS	THNCGHHKDAGVICS	0,3429
**chr4:g.153636481C>T**	YPGFNPFRAYMNLDI	YPGFNPFCAYMNLDI	0,3426
**chr11:g.58954809C>T**	DSYTNVYRMFSKEPQ	DSYTNVYCMFSKEPQ	0,3426
**chr8:g.113098901G>A**	IISSPSFPNEYHNNA	IISSPSFSNEYHNNA	0,3426
**chrX:g.74742674C>T**	FLPPARKRKSKLGNR	FLPPARKQKSKLGNR	0,3424
**chr7:g.144050939G>A**	EINHFFCEILSVLKL	EINHFFCKILSVLKL	0,3422
**chr17:g.11763546G>A**	IPADCPKEIYEHYFV	IPADCPKKIYEHYFV	0,3419
**chr2:g.21013214G>A**	DHFSLRARYHMKADS	DHFSLRACYHMKADS	0,3418
**chr10:g.95069350G>A**	LSFMLLFSLWRQSCR	LSFMLLFLLWRQSCR	0,3417
**chr20:g.33379553G>A**	KTKQGGLRFAHLLDQ	KTKQGGLCFAHLLDQ	0,3416
**chr4:g.174677184G>A**	IVILSWVSFWINMDA	IVILSWVLFWINMDA	0,3412
**chr4:g.163325948G>A**	YVCFDQFPSDSHRLS	YVCFDQFSSDSHRLS	0,3409
**chr3:g.155494221C>T**	LNYQSEGRMMQLNRA	LNYQSEGQMMQLNRA	0,3408
**chr7:g.6692311G>A**	ESLYQKIRILEKPFE	ESLYQKICILEKPFE	0,3404
**chr16:g.55823605C>T**	GLALAAHENVVVVTI	GLALAAHKNVVVVTI	0,3403
**chr7:g.142054866C>T**	DVLIKKNPFGIEIRR	DVLIKKNLFGIEIRR	0,3399
**chr17:g.10540036C>T**	DILGFTNEEKVSIYK	DILGFTNKEKVSIYK	0,3395
**chr10:g.24595140G>A**	RKEDPLDRLKTLKRL	RKEDPLDCLKTLKRL	0,3393

**chr3:g.108353708G>A**	RRVSLLDEGIYTCYV	RRVSLLDKGIYTCYV	0,3387
**chr11:g.26712676G>A**	LRFNKPVRYAATVIY	LRFNKPVCYAATVIY	0,3385
**chr3:g.109328939G>A**	LLPASNFPPPHLEDN	LLPASNFSPPHLEDN	0,3379
**chr15:g.83264083C>T**	IKHKCTIEGCNMVFS	IKHKCTIKGCNMVFS	0,3379
**chr12:g.31982988C>T**	EVLATCLSLWKKQPS	EVLATCLFLWKKQPS	0,3379
**chr17:g.64561537G>A**	KRLMIKFRGEEGLDY	KRLMIKFCGEEGLDY	0,3377
**chr3:g.165065320G>A**	EQVHKRFRHDLSWKT	EQVHKRFCHDLSWKT	0,3374
**chr19:g.43195196C>T**	DVLTFTCEPKSKNYT	DVLTFTCKPKSKNYT	0,3374
**chr16:g.81270255G>A**	FHHVNAYEEDGCIVF	FHHVNAYKEDGCIVF	0,3373
**chrX:g.108695336C>T**	GSCLEEFRSAPFIEC	GSCLEEFCSAPFIEC	0,3369
**chr3:g.148882526C>T**	NRMWRKNRSKNQNSK	NRMWRKNCSKNQNSK	0,3365
**chr8:g.72936404C>T**	AMGIMIFSSLVFFAE	AMGIMIFFSLVFFAE	0,3365
**chr1:g.78946388C>T**	NLTQSCGENANCTNT	NLTQSCGKNANCTNT	0,3365
**chr1:g.74631757G>A**	TWRRRRFRPTTAPNG	TWRRRRFCPTTAPNG	0,3364
**chr14:g.78645206G>A**	PIQSSSDEITLSFKT	PIQSSSDKITLSFKT	0,3364
**chr1:g.201383118C>T**	KRHLFEKELAGQSRA	KRHLFEKKLAGQSRA	0,3363
**chr15:g.71012885C>T**	SSELPQYRLISILGD	SSELPQYCLISILGD	0,3362
**chr9:g.116440658C>T**	HHYNSHYEKFGDFVW	HHYNSHYKKFGDFVW	0,3362
**chr1:g.152358782C>T**	LSKGELKELLEKELH	LSKGELKKLLEKELH	0,3361
**chr2:g.228017604C>T**	RLKASSCESIPEEDS	RLKASSCKSIPEEDS	0,3358
**chr2:g.1910298C>T**	EKLAKAQEKHQSCDV	EKLAKAQKKHQSCDV	0,3352
**chr3:g.77493309C>T**	EEEAVEFRCQVQGDP	EEEAVEFCCQVQGDP	0,3351
**chr3:g.56434932G>A**	PRLPRSPRLGHRRTS	PRLPRSPCLGHRRTS	0,3347
**chr7:g.37741192G>A**	SKVAFYNEIFLSVTA	SKVAFYNKIFLSVTA	0,3345
**chrX:g.106036817G>A**	SAALVMLSFGACCST	SAALVMLFFGACCST	0,3344
**chr11:g.56360793C>T**	HFYCDNVPLLALSCS	HFYCDNVSLLALSCS	0,3342
**chr20:g.2602176C>T**	IHLTRFLRVLANFLI	IHLTRFLCVLANFLI	0,3328
**chr1:g.33725442G>A**	VYHGTQVPQFLISTS	VYHGTQVSQFLISTS	0,3328
**chr17:g.41467034C>T**	AFNGNEKETMQFLND	AFNGNEKKTMQFLND	0,3321
**chr7:g.82915589G>A**	HQIKQEFRRGTESLD	HQIKQEFCRGTESLD	0,332
**chr1:g.158420043G>A**	SSQDALISVSYTIIT	SSQDALILVSYTIIT	0,3318
**chr8:g.22148519G>A**	AQAVKHFRAGRDSYL	AQAVKHFCAGRDSYL	0,3314
**chrY:g.5057196C>T**	TTGARIDREKLCAGI	TTGARIDCEKLCAGI	0,3311
**chr1:g.190226184G>A**	CHCGPKFPECNCPSM	CHCGPKFSECNCPSM	0,3307
**chr10:g.20859799C>T**	YKEKFDNEMKDKKHH	YKEKFDNKMKDKKHH	0,3301
**chr1:g.56696086C>T**	YIPEYLNRSVATLLM	YIPEYLNCSVATLLM	0,33
**chr2:g.165113898C>T**	GKIWWNLRKTCYSIV	GKIWWNLQKTCYSIV	0,3296
**chr19:g.8090123C>T**	EGWGDPCELCPQEGS	EGWGDPCKLCPQEGS	0,3292
**chr10:g.54023128G>A**	GNFNNLFRITSNGSI	GNFNNLFCITSNGSI	0,3292
**chrX:g.87622265G>A**	FLLLPANEISKLLCS	FLLLPANKISKLLCS	0,3291
**chr7:g.142054883C>T**	NPFGIEIRRKSTGTI	NPFGIEICRKSTGTI	0,3287
**chr6:g.29396724G>A**	LNTTSVTEFLLLGVT	LNTTSVTKFLLLGVT	0,3285
**chrX:g.78755275C>T**	LTCISVDRFLAIVYP	LTCISVDCFLAIVYP	0,3275

**chr5:g.137753490G>A**	GPFNQEFRDLLLNDW	GPFNQEFCDLLLNDW	0,3271
**chr4:g.46303573C>T**	TAHFHLKRKIGYFVI	TAHFHLKKKIGYFVI	0,3267
**chr12:g.85980131G>A**	PEDRVKFRSKQNVDY	PEDRVKFCSKQNVDY	0,3262
**chr1:g.176957704G>A**	NFRCISDRKLDSTGC	NFRCISDCKLDSTGC	0,3254
**chr7:g.87550003C>T**	INVRFLREIIGVVSQ	INVRFLRKIIGVVSQ	0,3249
**chrX:g.151700043C>T**	SVLKRFPRANEFLEE	SVLKRFPCANEFLEE	0,3244
**chr11:g.5047178C>T**	NLVLIGISYVYILRA	NLVLIGILYVYILRA	0,3243
**chr5:g.157211329C>T**	LYVFAPDRESRQRWV	LYVFAPDCESRQRWV	0,3239
**chr1:g.177280461G>A**	QSLLYCGESTFPGTF	QSLLYCGKSTFPGTF	0,3238
**chr1:g.220141882C>T**	EILDVIVRSFAPILP	EILDVIVCSFAPILP	0,3237
**chr10:g.82951508C>T**	QRQVLSISCIIFGIV	QRQVLSILCIIFGIV	0,3236
**chr1:g.111454296C>T**	PEYGGKVRYGLIPEE	PEYGGKVCYGLIPEE	0,3234
**chr15:g.71898626G>A**	SLELLKVRSLGGISP	SLELLKVCSLGGISP	0,3232
**chr1:g.18840330G>A**	HFQTPIVRSAGGPMC	HFQTPIVCSAGGPMC	0,3228
**chr1:g.241098711G>A**	EKNGIPIRTVKSFLS	EKNGIPICTVKSFLS	0,3228
**chr2:g.166073486C>T**	PDKKDDDENGPKPNS	PDKKDDDKNGPKPNS	0,3227
**chr19:g.44086973C>T**	FTENSKLRFHQRIHT	FTENSKLCFHQRIHT	0,3217
**chr1:g.158579821C>T**	INQTILKEFILVGFS	INQTILKKFILVGFS	0,3213
**chr22:g.15528438C>T**	WYVSSTVPKMLVNFL	WYVSSTVSKMLVNFL	0,321
**chr14:g.19876917C>T**	CFIVLFNSYVIVLVT	CFIVLFNLYVIVLVT	0,3204
**chr11:g.4848629G>A**	CLHPDVARLACPEAW	CLHPDVACLACPEAW	0,3193
**chr1:g.93589377G>A**	RDGDFLVRDSLSSPG	RDGDFLVCDSLSSPG	0,3192
**chr10:g.112417867C>T**	DSLGGRVRVIVTGAA	DSLGGRVCVIVTGAA	0,3191
**chr1:g.222543630G>A**	RKFYIYYSCLDKKKV	RKFYIYYLCLDKKKV	0,3176
**chr19:g.51769424C>T**	SFFICWFPFQLVALL	SFFICWFSFQLVALL	0,3164
**chr2:g.166199061G>A**	SGEMDSLRSQMEERF	SGEMDSLCSQMEERF	0,3157
**chr7:g.137552495G>A**	VMLLTYKSIPMQVDG	VMLLTYKFIPMQVDG	0,3148
**chr2:g.211623993G>A**	APNQAQLRILKETEL	APNQAQLCILKETEL	0,3147
**chr6:g.72258226C>T**	SRSVSPHRGNDQGKP	SRSVSPHCGNDQGKP	0,3146
**chr12:g.32821502C>T**	QDVPMPEEKSNPKGV	QDVPMPEKKSNPKGV	0,3142
**chr3:g.123700608G>A**	SPQQVDFRSVLAKKG	SPQQVDFCSVLAKKG	0,314
**chr6:g.55874706C>T**	ERREIQREILSILGL	ERREIQRKILSILGL	0,3134
**chr16:g.80549576C>T**	MKAKSTVRDIDPQND	MKAKSTVCDIDPQND	0,3126
**chr10:g.119859121G>A**	PNSFVKFRCMIQDMF	PNSFVKFCCMIQDMF	0,3126
**chr2:g.211947535G>A**	LENLRIIRGTKLYED	LENLRIICGTKLYED	0,3119
**chr5:g.26915774G>A**	EKSLYILRAKAIDRK	EKSLYILCAKAIDRK	0,3116
**chr3:g.98169063C>T**	LATMAYDRYVAICKP	LATMAYDCYVAICKP	0,3113
**chr15:g.71813596G>A**	ALAVDPTEFACMKAL	ALAVDPTKFACMKAL	0,3111
**chr17:g.10543769G>A**	DQIISANPLLEAFGN	DQIISANLLLEAFGN	0,3111
**chr12:g.46776966G>A**	MQTVSNISITGMLVM	MQTVSNIFITGMLVM	0,311
**chr11:g.67455721C>T**	SRQSHRHRPDSLHDA	SRQSHRHCPDSLHDA	0,3106
**chr14:g.70167995G>A**	LTLMALGSSAPEILL	LTLMALGFSAPEILL	0,3101
**chr4:g.69214260G>A**	VFADAVFPCGELLAA	VFADAVFSCGELLAA	0,3093

**chr7:g.50399977G>A**	ASYEKENEMMKSHVM	ASYEKENKMMKSHVM	0,3092
**chr2:g.27601392C>T**	HHCGKQLRSLAGMKY	HHCGKQLCSLAGMKY	0,3088
**chr1:g.247531971C>T**	CIDHFFCEMPLIMQL	CIDHFFCKMPLIMQL	0,308
**chr20:g.54591666G>A**	PNLDVHGECALQITY	PNLDVHGKCALQITY	0,3078
**chr4:g.150193204C>T**	ACGPEKFRYAQDDFV	ACGPEKFCYAQDDFV	0,3066
**chr5:g.41012687C>T**	EILMFLEEMLDGLES	EILMFLEKMLDGLES	0,3064
**chr7:g.100755252G>A**	CPPNARYESCACPAS	CPPNARYKSCACPAS	0,3063
**chr6:g.54326863C>T**	LEDGFKFRLGTLPPS	LEDGFKFCLGTLPPS	0,3062
**chr9:g.117039911G>A**	LTLIAVSSCILAMVC	LTLIAVSFCILAMVC	0,3059
**chr6:g.136698529G>A**	ICLPLVDRFIQLLKV	ICLPLVDCFIQLLKV	0,3055
**chr18:g.3126853G>A**	ITCLESFRDSMVLGW	ITCLESFCDSMVLGW	0,3055
**chr6:g.63280493G>A**	AGLLKHSAALSCLMA	AGLLKHSVALSCLMA	0,3049
**chr3:g.47428577G>A**	LLAVDVFRSPLSRAF	LLAVDVFCSPLSRAF	0,3047
**chr3:g.98149606C>T**	AFVDALLSSSVTLKM	AFVDALLLSSVTLKM	0,3045
**chr2:g.161894712C>T**	VAYKAKDRNDLVSGI	VAYKAKDCNDLVSGI	0,3029
**chr13:g.37692212G>A**	FIIGLLFPVFSVCYL	FIIGLLFSVFSVCYL	0,3027
**chr2:g.165313720C>T**	WAFLSLFRLMTQDFW	WAFLSLFCLMTQDFW	0,3024
**chr10:g.70708725C>T**	VVDDSVVRFHGKEHV	VVDDSVVCFHGKEHV	0,3022
**chr3:g.50256262C>T**	QQDVLRTRVKTTGIV	QQDVLRTCVKTTGIV	0,3019
**chr9:g.104598876C>T**	IINHFTCEILAVMKL	IINHFTCKILAVMKL	0,3017
**chr3:g.39265641G>A**	FSHCCLNPLIYAFAG	FSHCCLNLLIYAFAG	0,3014
**chr1:g.173517575C>T**	LSCLLKREIEYTVIC	LSCLLKRKIEYTVIC	0,3013
**chr13:g.23332514G>A**	SAEKREFRFQLRGVA	SAEKREFCFQLRGVA	0,3012
**chr8:g.107958215G>A**	GTCSRNNRTCGFKWG	GTCSRNNCTCGFKWG	0,3011
**chr15:g.30913938C>T**	SRILLLFSLTDSMED	SRILLLFLLTDSMED	0,3005
**chr15:g.50248313G>A**	IPLSRRFRSVKLWFV	IPLSRRFCSVKLWFV	0,2991
**chr8:g.94668086G>A**	CPITGGKEGILFVTY	CPITGGKKGILFVTY	0,2989
**chr16:g.70882731G>A**	VGRQAKARFKISNVG	VGRQAKACFKISNVG	0,2983
**chr4:g.6863707C>T**	ISPRTHFRPISASEL	ISPRTHFCPISASEL	0,2976
**chr17:g.75812164C>T**	TERRYHLRYYKTGIC	TERRYHLCYYKTGIC	0,2969
**chr18:g.5291964G>A**	VIEIDFLRSDIFEEV	VIEIDFLCSDIFEEV	0,2969
**chr11:g.4914799G>A**	GFMYLLFPPVMNPIV	GFMYLLFSPVMNPIV	0,2969
**chr2:g.137618415G>A**	WSTCQLSENAPCGQG	WSTCQLSKNAPCGQG	0,2966
**chr4:g.79407213G>A**	AIAGAVIRWLRDNLG	AIAGAVICWLRDNLG	0,2958
**chr17:g.10445044G>A**	TVKDLQLRLDEAEQL	TVKDLQLCLDEAEQL	0,2954
**chr10:g.67180358G>A**	IAECNAIRQALQDLL	IAECNAICQALQDLL	0,2952
**chr1:g.56512053G>A**	AFYTGLSRVSDHKHH	AFYTGLSCVSDHKHH	0,2947
**chr8:g.3359230G>A**	FSCFLGYRLEGATKL	FSCFLGYCLEGATKL	0,2947
**chr10:g.17043911G>A**	DEKPPLIRSSGDSMF	DEKPPLICSSGDSMF	0,2945
**chr9:g.23704942G>A**	YGRIITSRILVDQVT	YGRIITSCILVDQVT	0,2935
**chr17:g.11923879G>A**	VSLGQGQEVVAEAAL	VSLGQGQKVVAEAAL	0,2924
**chr6:g.151593221C>T**	ARTEQLVRLESNAVI	ARTEQLVCLESNAVI	0,2917
**chr8:g.86229754G>A**	VSILSFISLTGVVFL	VSILSFIFLTGVVFL	0,2912

**chr16:g.20032148G>A**	LRRKSNFRLRGYSTG	LRRKSNFCLRGYSTG	0,2912
**chr1:g.200120892C>T**	QELVAKLRSLQFDQR	QELVAKLCSLQFDQR	0,2902
**chr11:g.123940266C>T**	CFLVLLGSYTALLVM	CFLVLLGLYTALLVM	0,2901
**chr12:g.125644115G>A**	SIFVNGKEMKSKVDT	SIFVNGKKMKSKVDT	0,2897
**chr12:g.21178635C>T**	VFMGNMLRGIGETPI	VFMGNMLCGIGETPI	0,2896
**chr3:g.167697033G>A**	NFTESLLRMAADDVE	NFTESLLCMAADDVE	0,2888
**chr6:g.101818457C>T**	ITDSLSNRSLIVTTI	ITDSLSNCSLIVTTI	0,2883
**chr8:g.15748437C>T**	SFLLSIFRSKYHGYP	SFLLSIFCSKYHGYP	0,2875
**chr19:g.51142739C>T**	DAGRYFFRMEKGNIK	DAGRYFFCMEKGNIK	0,2862
**chr3:g.38847035G>A**	LPMVSEDRLHCMDIL	LPMVSEDCLHCMDIL	0,286
**chr5:g.116012428C>T**	NIYSTIIRENVLALQ	NIYSTIICENVLALQ	0,2852
**chr11:g.121574273C>T**	CDLDTQFRCQESGTC	CDLDTQFCCQESGTC	0,2848
**chr11:g.123943058G>A**	SSIFLYIRMSEAQSK	SSIFLYICMSEAQSK	0,2847
**chr19:g.51746508G>A**	LTLPVIIRVTTVPGK	LTLPVIICVTTVPGK	0,2845
**chrX:g.143629380C>T**	ELKSLKNEILCPKLL	ELKSLKNKILCPKLL	0,2843
**chr6:g.131652568C>T**	IWMCNKFRCGETRLE	IWMCNKFCCGETRLE	0,2832
**chr9:g.32632918G>A**	WVLKSDFRLPTEEEI	WVLKSDFCLPTEEEI	0,2831
**chr10:g.37219040G>A**	ENCMLKKEIAMLKLE	ENCMLKKKIAMLKLE	0,2827
**chr11:g.121570183C>T**	TCLRNQYRCSNGNCI	TCLRNQYCCSNGNCI	0,2815
**chr7:g.75954096C>T**	MTDMILFSLIVGLLT	MTDMILFLLIVGLLT	0,2795
**chr4:g.42962946C>T**	VIYTTCLRVVRTTFE	VIYTTCLCVVRTTFE	0,2792
**chr16:g.70952513G>A**	IKPGKSVRGSVVITK	IKPGKSVCGSVVITK	0,278
**chr12:g.52792340G>A**	VLSMDNNRSLDLDSI	VLSMDNNCSLDLDSI	0,2773
**chr1:g.157547108G>A**	TLTCKGFRFYSPQKT	TLTCKGFCFYSPQKT	0,2765
**chr9:g.125149801G>A**	SAPNYCYRCGNIASI	SAPNYCYCCGNIASI	0,2743
**chr1:g.81969239C>T**	TWVGIVISLVCLAIC	TWVGIVIFLVCLAIC	0,273
**chr14:g.94288428G>A**	STFDKNFRCHVLKLP	STFDKNFCCHVLKLP	0,2728
**chr17:g.49045024C>T**	ETPDSKVRMVIITGP	ETPDSKVCMVIITGP	0,2725
**chr7:g.143321747C>T**	PEMKTILRGVVLKEY	PEMKTILCGVVLKEY	0,2702
**chr11:g.24914509C>T**	QAQLKELRYGKKDLL	QAQLKELCYGKKDLL	0,2666
**chr8:g.24500858C>T**	GVLILLVRYRKCIKL	GVLILLVCYRKCIKL	0,2644
**chr13:g.37637598G>A**	KQDISSFRFEVLGLL	KQDISSFCFEVLGLL	0,2627
**chr4:g.122353074C>T**	CFHGPNFRSKSWALF	CFHGPNFCSKSWALF	0,2613
**chr16:g.14878784C>T**	SLSGGLFRSNLLTQD	SLSGGLFCSNLLTQD	0,2598
**chr2:g.37037998G>A**	GATTSTIRSSCLVGC	GATTSTICSSCLVGC	0,2597
**chr3:g.11026264C>T**	IIVCCINSCTSMFAG	IIVCCINLCTSMFAG	0,2577
**chr12:g.45836908C>T**	AANRTCLRFLLLSAH	AANRTCLCFLLLSAH	0,2568
**chr2:g.218135765C>T**	AFIGQKFRHGLLKIL	AFIGQKFCHGLLKIL	0,2537
**chr13:g.85795938G>A**	PLTHLDLRGNQLQTL	PLTHLDLCGNQLQTL	0,2529
**chr8:g.24442511C>T**	VEGQQLVRPKKLPLI	VEGQQLVCPKKLPLI	0,2096
